# Reactions of biologically inspired hydride sources with B(C_6_F_5_)_3_

**DOI:** 10.1098/rsta.2017.0009

**Published:** 2017-07-24

**Authors:** Lewis C. Wilkins, Nicolò Santi, Louis Y. P. Luk, Rebecca L. Melen

**Affiliations:** School of Chemistry, Cardiff University, Main Building, Park Place, Cardiff CF10 3AT, UK

**Keywords:** frustrated Lewis pair, hydrogenation, dehydrogenation, boron, dihydropyridine

## Abstract

The combination of 1-benzyl-1,4-dihydropyridines with the strong Lewis acid, B(C_6_F_5_)_3_, generates a stable pyridinium borohydride species in high yields (94%) in as little as 10 min. This use of biologically inspired hydride sources further builds on the recent work of new hydride donors in the formation of borohydrides. When functionalizing the dihydropyridine with an amide or carboxylic acid moiety, a disproportionation reaction composed of a series of protonation/reduction steps is observed upon the addition of B(C_6_F_5_)_3_. As a result, one equivalent of dihydropyridine undergoes net hydrogenation, whereas the other is dehydrogenated yielding the pyridinium counterpart in a transfer hydrogenation-type mechanism.

This article is part of the themed issue ‘Frustrated Lewis pair chemistry’.

## 1. Introduction

In recent years, the field of frustrated Lewis pairs has seen vast developments in a number of applications as a way of inducing alternative reactivity, sometimes counter to what is the ‘traditional’ pathway of Lewis acid/base reactions [1]. While mixtures of conventional Lewis acids and Lewis bases result in adduct formation, which is a ubiquitous motif in organic and organometallic chemistry, a frustrated system is achieved when the steric bulk about the lone pair donor and acceptor is increased, thus precluding adduct formation. The development of frustrated Lewis pairs has led to great advancements in small molecule activation, particularly in molecular hydrogen activation through heterolytic H─H bond cleavage [1–5]. Alternative applications have focused on the utilization of Lewis acids, mainly but not limited to the archetypal B(C_6_F_5_)_3_, to effect other transformations through 1,*n*-carboborations [6–8], hydrosilylations [9–11], hydroborations [12,13], cyclizations [14–18] and benzannulations [19,20], among others [21,22].

**Scheme 1.**
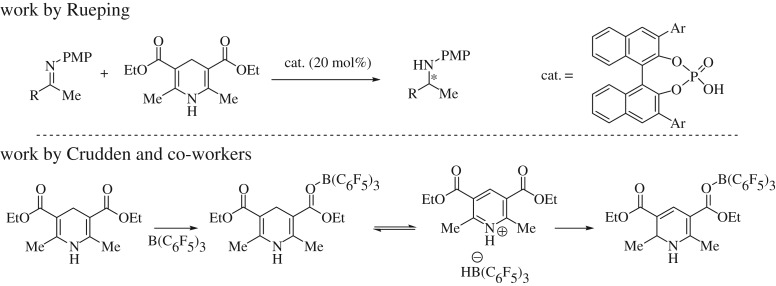
Recent work using Hantzsch esters as hydrogen storage mediums (PMP, *para*-methoxyphenyl).

The Hantzsch ester is a good hydrogen storage medium due to its ease of manipulation compared with molecular hydrogen, and is able to provide both the hydridic and the protic components for reduction when combined with a catalyst [23]. Many disciplines have investigated differing methods of accessing this hydrogen storage medium with the majority focusing on organocatalytic routes, such as the work of Rueping *et al*. [24], although transition metals have also been used [25]. Recent work by Crudden and co-workers [26] focused on the exposition of B(C_6_F_5_)_3_ to Hantzsch esters in a bid to form borohydrides from organic sources (scheme 1).

**Figure 1.**
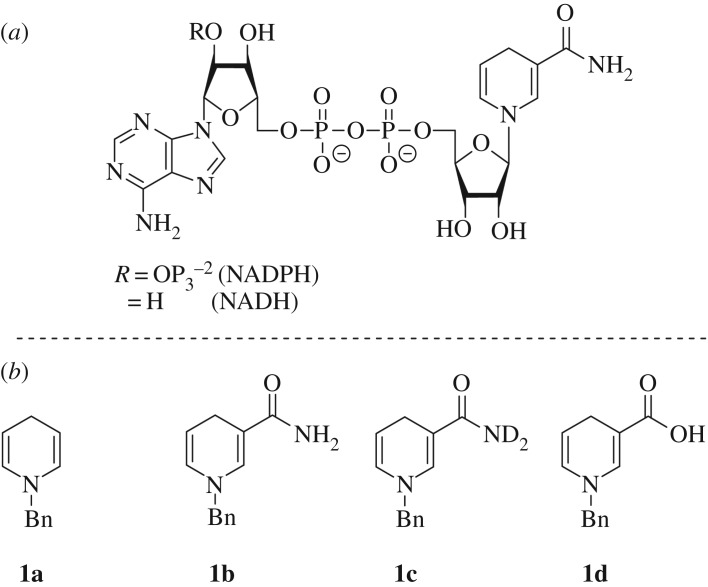
Structure of NAD(P)H (*a*) and 1,4-dihydropyridine reagents investigated in this work (*b*).

With borohydrides being pre-eminent in the reduction of various unsaturated frameworks, novel borohydride moieties as well as synthetic routes are highly desirable to synthetic organic chemists. Indeed, there are few examples of organic hydride donors being used to form borohydrides; furthermore, there is little precedence for the utilization of biological analogues as hydride sources in conjunction with Lewis acidic boranes [27]. As 1-benzyl-1,4-dihydronicotinamide (BNAH, **1b**) is considered a close artificial analogue of the biologically sourced nicotinamide adenine dinucleotide (phosphate) (NAD(P)H), it may provide a good basis for new hydride sources for facile borohydride synthesis [28]. Initial investigations focused on the simplest 1-benzyl-1,4-dihydropyridine featuring only the hydridic component, with further reactivity directed at heterocycles functionalized with amide or carboxylic acid groups at the C-3 position (figure 1).

Herein, we explore the uses of such hydride sources in conjunction with the strong Lewis acid B(C_6_F_5_)_3_ to forge new ways of generating borohydrides from naturally inspired supplies and comparing their reactivity to that of Hantzsch esters reported previously [26].

## 2. Experimental methods

### (a) General

With the exception of the starting materials, all reactions and manipulations were carried out under an atmosphere of dry, O_2_-free nitrogen using standard double-manifold techniques with a rotary oil pump. A nitrogen-filled glove box (MBraun) was used to manipulate solids including the storage of starting materials, room temperature reactions, product recovery and sample preparation for analysis. All solvents (CH_2_Cl_2_, hexane, pentane) were dried by employing a solvent purification system MB SPS-800 and stored under a nitrogen atmosphere. Deuterated solvents were distilled and/or dried over molecular sieves before use. Chemicals were purchased from commercial suppliers and used as received.

### (b) Dihydropyridine synthesis

1-Benzyl-1,4-dihydropyridine (**1a**): The compound **1a** was synthesized in accordance with previously outlined procedures [29]. Pyridine (2.0 ml, 25.3 mmol) was dissolved in acetonitrile (30 ml), then benzyl bromide (3.0 ml, 25.3 mmol) was added. The reaction mixture was stirred under reflux at 80°C for 12 h. The solution was then cooled and diethyl ether (50 ml) was added to precipitate the crude product. After filtering off the supernatant and washing the solid with diethyl ether (3 × 10 ml), the bromide salt was obtained as a yellow solid (1.9 g, 7.6 mmol, 30%). A suspension of water (70 ml) and toluene (50 ml) containing sodium dithionite (10.0 g, 60.0 mmol) and sodium carbonate (8.0 g, 70.0 mmol) was stirred vigorously under a nitrogen atmosphere and was heated to 100°C. 1-Benzyl pyridinium bromide (2.5 g, 10.0 mmol) dissolved in water (100 ml) was then added in small portions over a period of 10 min. After reflux for 10 min, the organic solution was separated and was washed with saturated sodium bicarbonate solution (3 × 10 ml), followed by water (3 × 10 ml) with the organic layer being dried with Na_2_SO_4_. The solvent was removed under reduced pressure to yield the product as a yellow/red oil (0.63 g, 3.7 mmol, 37%).

1-Benzyl-1,4-dihydronicotinamide (**1b**): The compound **1b** was synthesized in accordance with previously outlined procedures [30]. To a solution of nicotinamide (4.9 g, 40.0 mmol) in a mixture of 1,4-dioxane (100 ml) and methanol (25 ml), benzyl bromide (4.8 ml, 40.0 mmol) was added. The reaction mixture was stirred under reflux at 80°C for 4 h. The solution was then cooled and 1,4-dioxane (50 ml) was added to precipitate the crude product. After filtering off the supernatant and washing the solid with 1,4-dioxane (3 × 10 ml), the bromide salt was obtained as a white solid (7.5 g, 25.7 mmol, 64%). 1-Benzyl-3-carbamoyl pyridinium bromide (0.40 g, 1.4 mmol) was dissolved in water (15 ml) and sodium bicarbonate (0.78 g, 9.4 mmol) was added. Under a nitrogen atmosphere, sodium dithionite (1.63 g, 9.4 mmol) was added in small portions over a period of 10 min. The reaction mixture was stirred at room temperature for 3 h in the dark, during which time the solution turned from orange to yellow as the yellow product precipitated. The solid was filtered, washed with cold water (2 × 10 ml) and then dissolved in chloroform (20 ml). The organic phase was extracted with water (2 × 10 ml) and was subsequently dried over Na_2_SO_4_ and evaporated under vacuum to obtain a bright yellow powder (0.21 g, 1.0 mmol, 70%).

(^2^H,^2^H-N)-1-benzyl-1,4-dihydronicotinamide (**1c**): A small portion of compound **1b** was dissolved twice in methanol-d_4_, which was removed under reduced pressure, to yield a bright yellow powder of **1c**.

1-Benzyl-1,4-dihydronicotinic acid (**1d**): The compound **1d** was synthesized in accordance with previously outlined procedures [30]. Nicotinic acid (2.46 g, 20 mmol) was dissolved in a minimal amount of ethanol and diluted with acetonitrile (40 ml). Benzyl bromide (2.4 ml, 20 mmol) was then added and stirred under reflux at 80°C for 12 h. The solution was cooled and diethyl ether (50 ml) was added to precipitate the crude product. After filtering off the supernatant and washing the solid with diethyl ether (3 × 10 ml), the bromide salt was obtained as a white powder (3.5 g, 11.9 mmol, 60%). A suspension of 1-benzyl-3-carboxy pyridinium bromide (2.9 g, 10 mmol) in water (200 ml) and dichloromethane (100 ml) was cooled to 0°C and stirred under a nitrogen atmosphere. Sodium carbonate (6.4 g, 60 mmol) was added in small portions over a period of 10 min. Sodium dithionite (7 g, 40 mmol) was then added slowly over a period of 15 min. Stirring was continued over a period of 1 h under a nitrogen stream at 0°C. The organic layer was washed with water (3 × 100 ml), dried over Na_2_SO_4_ and the volatiles evaporated under vacuum to afford the yellow product (0.55 g, 2.6 mmol, 26%).

### (c) Product synthesis

1-Benzylpyridin-1-ium tris(pentafluorophenyl)hydroborate (**2**): Compound **1a** (17 mg, 0.1 mmol, 1 equiv) was dissolved in CDCl_3_ (0.5 ml, 0.2 M) to give a dark-red solution to which B(C_6_F_5_)_3_ (51 mg, 0.1 mmol, 1 equiv) was added. This was transferred to an NMR tube to monitor reaction progress. After 30 min, the reaction showed almost complete conversion to the hydridoborate product **2,** as observed by *in situ* NMR spectroscopy, at this point washing with cold pentane (3 × 2 ml) and removal of solvents *in vacuo* gave a dark-red viscous oil (63 mg, 92 µmol, 91%).

Pyridinium borates (**3**, **5** and **7**): The respective dihydropyridine reagent (0.2 mmol, 1 equiv) was dissolved in CDCl_3_ (0.5 ml, 0.2 M) to which B(C_6_F_5_)_3_ (102 mg, 0.2 mmol, 1 equiv) was added, yielding an orange solution. This was transferred to a J-Youngs NMR tube and heated for 12 h at 70°C. A crystalline solid precipitated out of solution which was isolated, with the mother liquor being retained for isolation of the corresponding tetrahydropyridines **4** and **6**. The remaining solid was washed with cold CH_2_Cl_2_ (2 × 2 ml) and dried *in vacuo* to garner a powdery solid (**3:** 63 mg, 87.0 µmol, 44%, **5**: 58 mg, 80.0 µmol, 40%, **7**: 52 mg, 71.7 µmol, 36%).

1-Benzyl-1,4,5,6-tetrahydropyridines (**4** and **6)**: The mother liquor from the formation of the pyridinium borates was retained with the solvents being subsequently removed under reduced pressure. The solid residue was then washed with cold pentane (2 × 2 ml) and dried *in vacuo* to yield a powdery solid (**4**: 61 mg, 83.4 µmol, 42%, **6**: 56 mg, 76.6 µmol, 38%).

### (d) Analysis

^1^H, ^13^C, ^11^B and ^19^F NMR spectra were recorded on a Bruker Avance 300, Bruker Avance II 400 or Bruker Ascend 500. Chemical shifts are expressed as parts per million (ppm, *δ*) downfield of tetramethylsilane and are referenced to CDCl_3_ (7.26/77.16 ppm) as internal standards. NMR spectra were referenced to CFCl_3_ (^19^F) and BF_3_·Et_2_O/CDCl_3_ (^11^B). The description of signals include s, singlet; d, doublet; t, triplet; q, quartet; pent., pentet; m, multiplet; br., broad. All coupling constants are absolute values and are expressed in Hertz (Hz). ^13^C NMR were measured as ^1^H decoupled. Yields are given as isolated yields. All spectra were analysed assuming a first-order approximation. IR-spectra were measured on a Shimadzu IRAffinity-1 photospectrometer. Mass spectra were measured on a Waters LCT Premier/XE or a Waters GCT Premier spectrometer. Crystallographic studies were undertaken of a single crystal mounted in paratone oil and studied on an Agilent SuperNova Dual three-circle diffractometer using Cu–Kα radiation and a CCD detector. Measurements were carried out at 150(2) K with temperatures maintained using an Oxford cryostream unless otherwise stated. Data were collected and integrated and data corrected for absorption using a numerical absorption correction based on Gaussian integration over a multi-faceted crystal model within CrysAlisPro [31]. The structure was solved by direct methods and refined against *F*^2^ within SHELXL-2013 [32]. A summary of crystallographic data are available as the electronic supplementary material and the structure deposited with the Cambridge Structural Database (CCDC deposition number 1532113). These data can be obtained free of charge from The Cambridge Crystallographic Data Centre via www.ccdc.cam.ac.uk/data_request/cif. Geometry optimizations through computational means were undertaken using the B3LYP hybrid functional [33,34] while instituting the 6-31G* [35] basis set using Gaussian 09 software [36] based on coordinates gained from experimental data. Images of these structures were generated through ORTEP-3 [37] and Mercury [38].

## 3. Results and discussion

### (a) Reactions with 1-benzyl-1,4-dihydropyridine

**Scheme 2.**
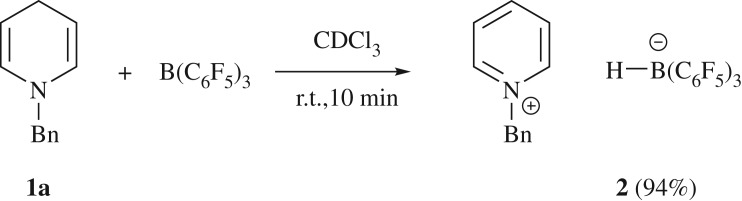
Synthesis of pyridinium borate **2**.

Initial investigations examined the reaction between the strong Lewis acid, B(C_6_F_5_)_3_, and the hydride donor 1,4-dihydropyridine **1a** featuring a benzyl functionalized tertiary amine. Upon combination, *in situ* multi-nuclear NMR spectroscopic data confirmed the formation of the aromatic pyridinium ring, with new characteristic resonances arising in the ^1^H NMR spectrum within 10 min at *δ* (ppm) = 8.65 (d, ^3^*J*_HH_ = 6.1 Hz, 2H, *o*-H), 8.43 (t, ^3^*J*_HH_ = 7.5 Hz, 1H, *p*-H) and 7.97 (t, ^3^*J*_HH_ = 7.1 Hz, 2H, *m*-H). This was accompanied by the tris(pentafluorophenyl)hydroborate anion, ([HB(C_6_F_5_)_3_]), producing a doublet in the ^11^B NMR spectrum at *δ* (ppm) = –25.2 ppm (scheme 2) [39]. These NMR studies indicate almost complete conversion to the corresponding pyridinium borate, which presented as an opaque red oil once volatiles were removed *in vacuo*. When less Lewis acidic boranes such as tris(2,4,6-trifluorophenyl) borane (B(C_6_H_2_F_3_)_3_) were used, hydride abstraction occurred, although several products were observed, with triphenyl borane (BPh_3_) showing little reactivity.

Crystallization of the pyridinium borate salt **2** for structural analyses was met with little success, as a bright red oil persisted, despite several crystallization techniques being used. The borohydride salt **2** that was formed was stable in solution for over 3 h at 70°C, in contrast with similar systems observed recently whereby the borohydride that is formed reduces the pyridinium at the *ortho*-position to generate the corresponding 1,2-dihydropyridine [26]. This subsequent reduction mechanism is driven by the reformation of the hard Lewis acid/base adduct between the carbonyl oxygen and the borane. In this work, however, the lack of carbonyl functionality, and slight steric congestion about the tertiary amine, enables the equilibrium to lie in favour of the pyridinium borohydride.

### (b) Reactions with functionalized 1,4-dihydropyridines

**Scheme 3.**
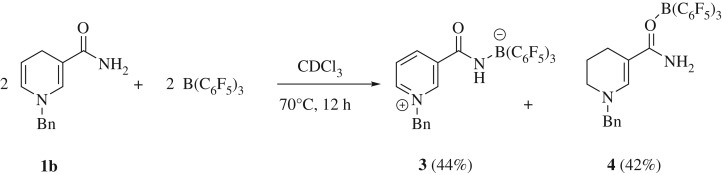
Reaction between **1b** and B(C_6_F_5_)_3_ to give 1 : 1 mixture of **3** and **4**.

A protic component was subsequently added to the benzyl dihydropyridine by including an amide functionality at the C-3 position. The stoichiometric reaction between the resulting 3,4-dihydropyridine **1b** (figure 1) and B(C_6_F_5_)_3_ yields a strong amide–boron adduct which is evident by a broad singlet at *δ* (ppm) = −2.4 in the ^11^B NMR spectrum, similar to observations in previous reports [14]. After 1 h, new resonances were observed in the ^1^H spectrum indicating the formation of the pyridinium salt, accompanied by a small sharp peak in the ^11^B NMR spectrum at *δ* (ppm) = −10.9 revealing the formation of a new borate species. This reaction was then heated to 70°C for 12 h at which point a white crystalline solid, suitable for structure determination by X-ray diffraction, precipitated out of solution in 44% yield. The isolated crystals were determined to be the new zwitterionic pyridinium borate **3** (scheme 3). It was initially thought that the reaction undergoes a dehydrogenative pathway, losing molecular H_2_, *en route* to the intramolecular pyridinium borate. Interestingly, closer examination of the mother liquor through NMR spectroscopic methods revealed another boron-containing compound to be present along with new aliphatic resonances in the ^1^H NMR spectrum. These new signals signify the partially hydrogenated dihydropyridine **4**, which was isolated as a pale green solid in 42% yield. Notably, the new triplets at *δ* (ppm) = 3.15 (t, ^3^*J*_HH_ = 4.9 Hz, 2H) and 2.11 (t, ^3^*J*_HH_ = 5.5 Hz, 2H) as well as the pentet at *δ* (ppm) = 1.91 (pent., ^3^*J*_HH_ = 5.4 Hz, 2H) can be assigned to newly formed *sp*^3^ centres of the heterocyclic structure with the *sp*^2^ vinylic proton presenting as a singlet at *δ* (ppm) = 7.92, shifted slightly downfield as a result of borane coordination to the conjugated system (scheme 3).

**Figure 2.**
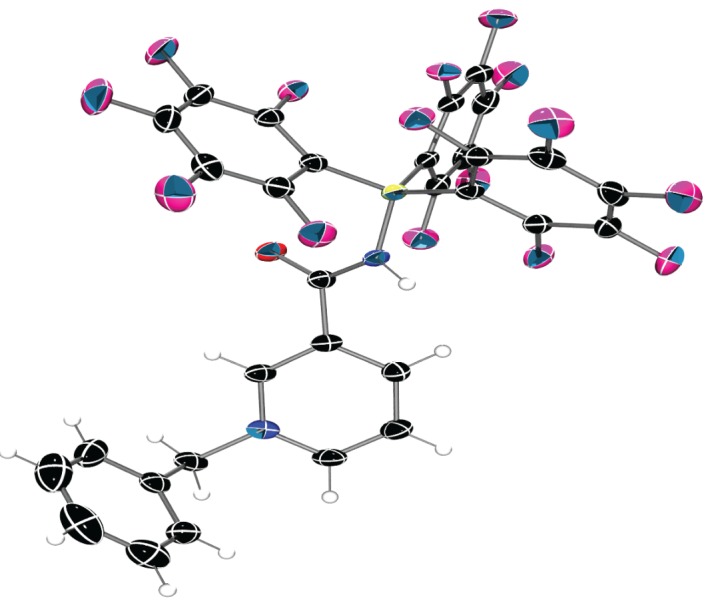
Solid-state structure of **3**. C, black; H, white; N, blue; O, red; B, yellow–green; F, pink. Thermal ellipsoids shown at 50% probability. Chloroform solvent molecule omitted for clarity.

**Figure 3.**
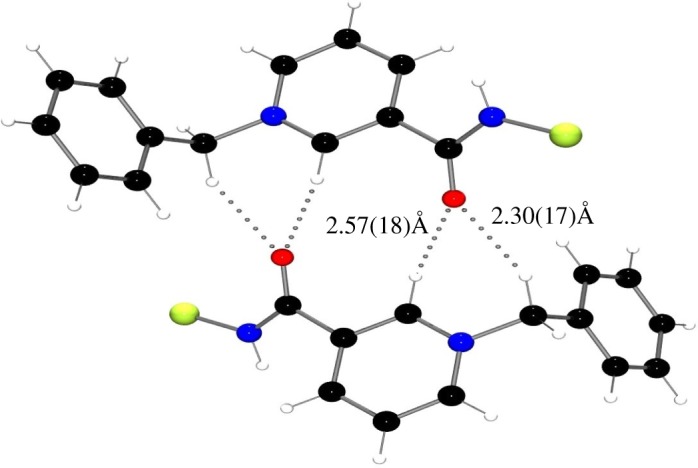
Solid-state packing of **3** depicting close intermolecular contacts. C_6_F_5_-groups omitted from boron atom for clarity. C, black; O, red; N, blue; B, yellow–green.

Structural analysis of **3** confirmed the formation of the pyridinium heterocycle with C–C bonds of the pyridinium measuring 1.369(5)–1.389(3) Å and C–N bonds measuring 1.345(3)–1.351(3) Å, both being consistent with average bond distances for aromatic nitrogen containing heterocycles (figure 2 and table 1) [40]. Additionally, the amide fragment lies out of plane of the pyridinium ring by 34.3(3)° in the solid state. This twist reduces orbital overlap, thus hindering the extent of conjugation, potentially arising from repulsive interactions brought about by the juxtaposition of the amide N–H and the aromatic C–H with a short interatomic H···H distance of 2.23(17) Å. In addition, when looking at the solid-state packing, short contacts are observed between the carbonyl oxygen and the electron-poor *o*-H of the pyridinium ring and benzylic proton (figure 3).

**Table 1.** Calculated and experimental bond distances for **3**. (Online version in colour.) 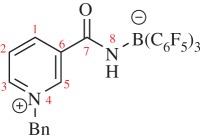

	bond lengths (Å)	
bond	experimental	calculated
C^1^–C^2^	1.389(4)	1.399
C^2^–C^3^	1.369(5)	1.382
C^3^–N^4^	1.345(3)	1.358
C^5^–N^4^	1.351(3)	1.348
C^5^–C^6^	1.379(4)	1.389
C^6^–C^1^	1.390(3)	1.398
C^6^–C^7^	1.516(4)	1.528
C^7^–O	1.228(3)	1.234
C^7^–N^8^	1.329(3)	1.333
N^8^–B	1.557(4)	1.570

**Figure 4.**
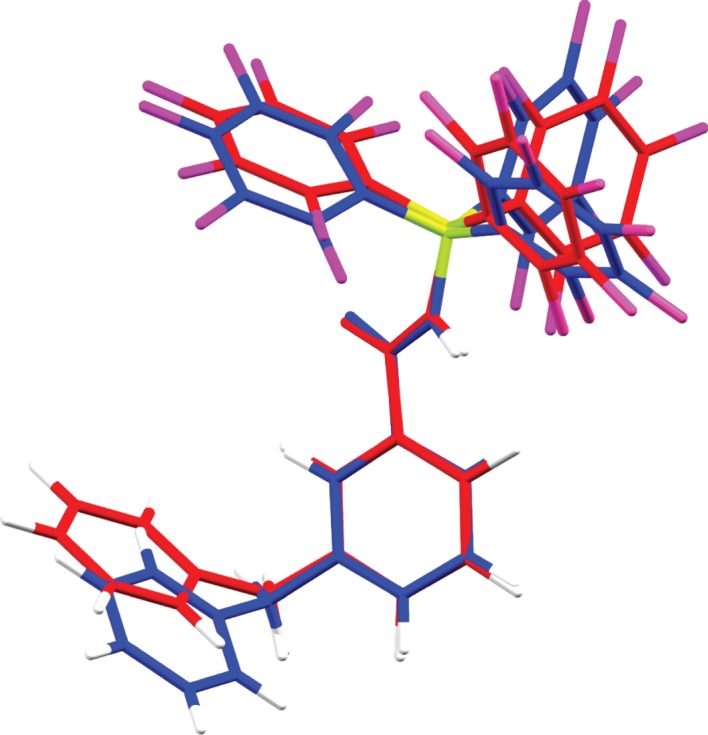
Overlay of solid-state structure (blue) and calculated structure (red) of **3** using B3LYP/6-31G* level of theory.

*In silico* studies were performed by way of geometry optimization of **3** with subsequent comparison of these values with that of the experimental values. While experimental bond distances of this structure agree well with the theoretical values calculated at the B3LYP/6-31G* level of theory (table 1), a slight rotation of the benzyl moiety was noted when comparing the overlaid structure (figure 4). A twist angle between the pyridinium ring and the phenyl ring of 64.6° in the calculated gas-phase structure was noted, compared with only 11.1(7)° in the experimental structure (figure 4).

**Scheme 4.**
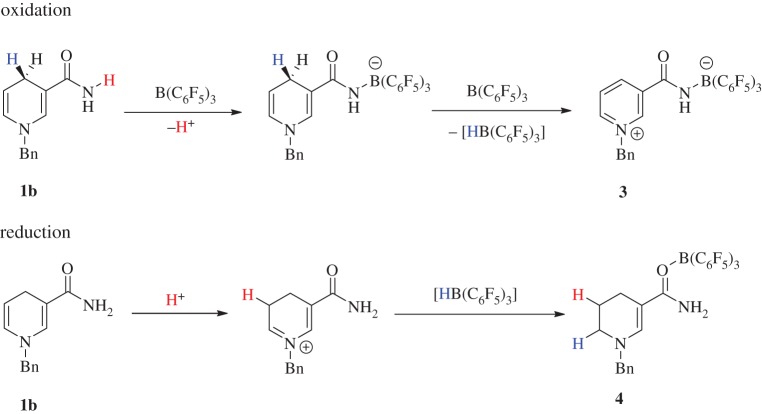
Proposed mechanistic pathway for the disproportionation reaction between **1b** and B(C_6_F_5_)_3_. (Online version in colour.)

Mechanistically, it was theorized that coordination of the Lewis acid to the amide oxygen acidifies the NH_2_ protons which can subsequently protonate the alkene moiety distal to the amide. Successive hydride migration yields the final zwitterionic pyridinium borate **3** and tetrahydropyridine borane adduct **4** in a 1 : 1 molar ratio (scheme 4). As half the borane source is simply sequestered as the Lewis acid/base adduct in **4**, using 0.5 equivalents should produce the borate salt and tetrahydropyridine. However, in the absence of excess borane, the reaction did not ensue, leading to the predomination of side reactions producing an intractable mixture of products. This difference in reactivity lends credence to the assumption that ‘free’ borane is necessary to act as a hydride shuttle in the formation of **3** and **4** from **1b** (scheme 4).

**Scheme 5.**
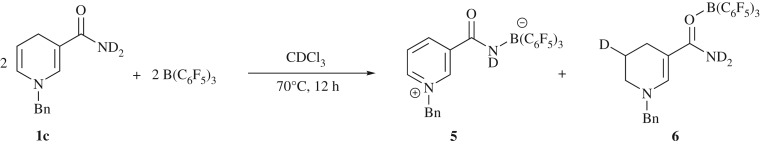
Deuterium labelling studies of **1c**.

Labelling studies were conducted to reveal the fate of the proton source during the course of the reaction. Compound **1b** was exposed to MeOD enabling deuterium exchange of the amide moiety to yield **1c**. This was then subjected to B(C_6_F_5_)_3_ in a 1 : 1 molar ratio in CDCl_3_ and heated to 70°C for 12 h, with the expected deuteration pattern being present in the final structures **5** and **6** (scheme 5). The previously observed triplet resonances now present as broad doublets at *δ* (ppm) = 3.15 (d, ^3^*J*_HH_ = 5.5 Hz, 2H, CH_2_) and 2.10 (d, ^3^*J*_HH_ = 6.3 Hz, 2H, CH_2_) while the pentet persisted, now with an integral of 1 at *δ* (ppm) = 1.91 (pent., ^3^*J*_HH_ = 6.2 Hz, 1H, CHD).

**Scheme 6.**
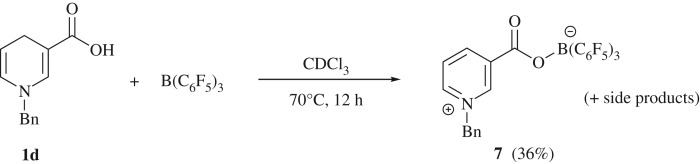
Reaction between 1-benzyl-1,4-dihydronicotinic acid **1d** and B(C_6_F_5_)_3_.

When this reaction was repeated in the presence of another unsaturated substrate, namely 4-fluorobenzaldehyde, interestingly no reduction to the alcohol was observed as the aldehyde proton signal remained unaltered. *In situ* NMR spectroscopy indicates the reaction proceeded to partially hydrogenate **1b** as before, albeit with a mixture of side-products being noted. The related 1-benzyl-1,4-dihydronicotinic acid **1d** was also tested, and, indeed, the formation of the pyridinium borate was noted in the ^1^H NMR spectrum as well as the oxygen-bound borate fragment presenting as a singlet in the ^11^B NMR spectrum at *δ* (ppm) = −4.0, with the product **7** being garnered in 36% yield (scheme 6). Unfortunately, the hydrogenated product proved difficult to isolate as the *in situ* spectroscopic data revealed a large number of side reactions also occurred.

## 4. Conclusion

This work has built upon an area of main group chemistry that has yet to be fully explored, specifically the generation of borohydrides from biologically inspired sources. It has been shown that the combination of the strong Lewis acid, B(C_6_F_5_)_3_, and unactivated dihydropyridines result in a thermally stable borohydride through hydride abstraction. In addition, functionalizing these dihydropyridines with amide and carboxylic acid units leads to unique reactivity when exposed to B(C_6_F_5_)_3_. The resulting products are in stark contrast with those observed previously whereby partial hydrogenation takes place in a disproportionation-type reaction with one equivalent of starting material being reduced by another equivalent via a series of protonation and hydride transfer mechanisms. These results show promise for future work in the synthesis of biologically sourced borohydrides, also showcasing how these methods can bridge several fields of synthetic chemistry, such as biochemical, inorganic, green and main group chemistry.

## Supplementary Material

experimental data dihydropyridine

## Supplementary Material

dihydropyridine crystal CIF

## Data accessibility

For electronic supplementary material on details of synthesis, calculations and crystallographic data of structures [1532113] and crystallographic data in CIF or other electronic format, see (doi:10.1098/rsta.2014.0049).
