# Supplementary material for: Reactions of biologically inspired hydride sources with B(C6F5)3
Source: Philos Trans A Math Phys Eng Sci. 2017 Jul 24;375(2101):20170009. doi: 10.1098/rsta.2017.0009 (PMC5540842; doi:10.1098/rsta.2017.0009)
Supplement: experimental data dihydropyridine [file rsta20170009supp1.pdf]

Supplementary material for [Author], [Year], [Title], *Phil. Trans. R. Soc. A.* doi: 10.1098/rsta.[paper ID in form xxxx.xxxx e.g. 10.1098/rsta.2014.0049]

Reactions of biologically inspired hydride sources with $B(C_6F_5)_3$

Lewis C. Wilkins, Nicolò Santi, Louis Y. P. Luk and Rebecca L. Melen*

School of Chemistry, Cardiff University, Main Building, Park Place, Cardiff, Wales, CF10 3AT, UK.

Contents

1. General remarks. 1
2. Synthesis of starting materials. 2
3. Synthesis of products. 4
4. NMR spectra. 7
4.1 NMR spectra of starting materials. 7
4.2 NMR spectra of products. 14
5. Theoretical considerations 38
6. Crystallographic studies. 39
7. References: 41

1. General remarks.

With the exception of the starting materials, all reactions and manipulations were carried out under an atmosphere of dry, O_2-free nitrogen using standard double-manifold techniques with a rotary oil pump. An argon- or nitrogen-filled glove box (MBraun) was used to manipulate solids including the storage of starting materials, room temperature reactions, product recovery and sample preparation for analysis. All solvents (CH_2Cl_2, hexane) were dried by employing a Grubbs-type column system (Innovative Technology) or a solvent purification system MB SPS-800 and stored under a nitrogen atmosphere. Deuterated solvents were distilled and/or dried over molecular sieves before use. Chemicals were purchased from commercial suppliers and used as received. ^{1}H, ^{13}C, ^{11}B and ^{19}F NMR spectra were recorded on a Bruker Avance II 400 or Bruker Ascend 500. Chemical shifts are expressed as parts per million (ppm, δ) and are referenced to $CDCl_3$ (7.26/77.16 ppm) as internal standards. NMR spectra were referenced to $CFCl_3$ (^{19}F) and $BF_3{\cdot}Et_2O/CDCl_3$ (^{11}B). The description of signals include: s = singlet, d = doublet, t = triplet, q = quartet, pent. = pentet, m = multiplet and br. = broad. All coupling constants are absolute values and are expressed in Hertz (Hz). ^{13}C NMR were measured as ^{1}H decoupled. Yields are given as isolated yields. All spectra were analysed assuming a first order approximation. IR-Spectra were measured on a Shimadzu IRAffinity-1 photospectrometer. Mass spectra were measured on a Waters LCT Premier/XE or a Waters GCT Premier spectrometer using ^{10}B isotope.

© The Authors under the terms of the Creative Commons Attribution License http://creativecommons.org/licenses/by/3.0/, which permits unrestricted use, provided the original author and source are credited.

2. Synthesis of starting materials.

*Synthesis of 1-benzyl-1,4-dihydropyridine (**1a**).*

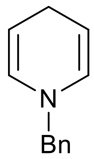

The compound **1a** was synthesised in accordance with previously outlined procedures.[1] Pyridine (2.0 mL, 25.3 mmol) was dissolved in acetonitrile (30 mL), then benzyl bromide (3.0 mL, 25.3 mmol) was added. The reaction mixture was stirred under reflux at 80 °C for 12 h. The solution was then cooled and diethyl ether (50 mL) was added to precipitate the crude product. After filtering off the supernatant and washing the solid with diethyl ether (3 x 10 mL), the bromide salt was obtained as a yellow solid (1.9 g, 7.6 mmol, 30%). A suspension of water (70 mL) and toluene (50 mL) containing sodium dithionite (10.0 g, 60.0 mmol) and sodium carbonate (8.0 g, 70.0 mmol) was stirred vigorously under a nitrogen atmosphere and was heated to 100 °C. 1-Benzyl pyridinium bromide (2.5 g, 10.0 mmol) dissolved in water (100 mL) was then added in small portions over a period of 10 mins. After reflux for 10 minutes, the organic solution was separated and was washed with saturated sodium bicarbonate solution (3 x 10 mL), followed by water (3 x 10 mL) with the organic layer being dried with Na_2SO_4. The solvent was removed under reduced pressure to yield the product as a yellow/red oil (0.63 g, 3.7 mmol, 37%). Analytical data agrees with previously reported values.[1] **^{1}H NMR** (400 MHz, $CDCl_3$, 298 K) δ/ppm: 7.33–7.23 (m, 5H, Ar-H), 5.73 (d, $^3J_{HH}$ = 7.9 Hz, 2H, NCH), 4.36–4.33 (m, 2H, =CH), 4.08 (s, 2H, N-CH_2), 2.95 (br. s, 2H, CH_2). **^{13}C NMR** (101 MHz, $CDCl_3$, 298 K), δ/ppm: 139.0 (s), 131.6 (s), 128.7 (s), 127.3 (s), 127.3 (s), 97.9 (s), 56.9 (s), 22.6 (s).

*Synthesis of 1-benzyl-1,4-dihydronicotinamide (**1b**).*

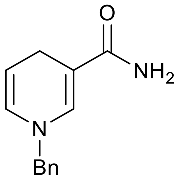

The compound **1b** was synthesised in accordance with previously outlined procedures.[2] To a solution of nicotinamide (4.9 g, 40.0 mmol) in a mixture of 1,4-dioxane (100 mL) and methanol (25 mL), benzyl bromide (4.8 mL, 40.0 mmol) was added. The reaction mixture was stirred under reflux at 80 °C for 4 h. The solution was then cooled and 1,4-dioxane (50 mL) was added to precipitate the crude product. After filtering off the supernatant and washing the solid with 1,4-dioxane (3 x 10 mL), the bromide salt was obtained as a white solid (7.5 g, 25.7 mmol, 64%). 1-benzyl-3-carbamoyl pyridinium bromide (0.40 g, 1.4 mmol) was dissolved in water (15 mL) and sodium bicarbonate (0.78 g, 9.4 mmol) was added. Under a nitrogen atmosphere, sodium dithionite (1.63 g, 9.4 mmol) was added in small portions over a period of 10 mins. The reaction mixture was stirred at room temperature for 3 hours in the dark, during which time the solution turned from orange to yellow as the yellow product precipitated. The solid was filtered, washed with cold water (2 x 10 mL) and then dissolved in chloroform (20 mL). The organic phase was extracted with water (2 x 10 mL) and was subsequently dried over Na_2SO_4 and evaporated under vacuum to obtain a bright yellow powder (0.21 g, 1.0 mmol, 70%). Analytical data agrees with previously reported values.[2] **^{1}H NMR** (400 MHz, $CDCl_3$, 298 K) δ/ppm: 7.35 (t, $^3J_{HH}$ = 7.2 Hz, 2H, Ar-H), 7.29 (d, $^3J_{HH}$ =

© The Authors under the terms of the Creative Commons Attribution License http://creativecommons.org/licenses/by/3.0/, which permits unrestricted use, provided the original author and source are credited.

7.2 Hz, 1H, Ar-H), 7.24 (d, $^3J_{HH}$ = 7.0 Hz, 2H, Ar-H), 7.15 (s, 1H, NCH), 5.73 (dq, $^3J_{HH}$ = 7.8 Hz, $^4J_{HH}$ = 1.6 Hz, 1H, NCH), 5.66 (br. s, 2H, NH_2), 4.74 (dt, $^3J_{HH}$ = 3.5 Hz, $^4J_{HH}$ = 8.0 Hz, 1H, =CH), 4.28 (s, 2H, N-CH_2), 3.16 (br. s, 2H, CH_2). **^{13}C NMR** (101 MHz, $CDCl_3$, 298 K), δ/ppm: 170.5 (s), 139.9 (s), 137.4 (s), 129.0 (s), 128.9 (s), 127.8 (s), 127.2 (s), 103.3 (s), 98.9 (s), 57.4 (s), 22.9 (s).

*Synthesis of (^{2}H,^{2}H-N)-1-benzyl-1,4-dihydronicotinamide (**1c**).*

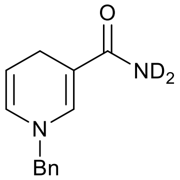

A small portion of compound **1b** was dissolved twice in methanol-d_4, which was removed under reduced pressure, to yield a bright yellow powder of **1c**. **^{1}H NMR** (400 MHz, $CDCl_3$, 298 K) δ/ppm: 7.36–7.28 (m, 3H, Ar-H), 7.23–7.21 (m, 2H, Ar-H), 7.12 (d, $^4J_{HH}$ = 1.4 Hz, 1H, NCH), 5.71 (dq, $^3J_{HH}$ = 8.2 Hz, $^4J_{HH}$ = 1.6 Hz, 1H, NCH), 4.72 (dt, $^3J_{HH}$ = 7.9 Hz, $^3J_{HH}$ = 3.4 Hz, =CH), 4.25 (s, 2H, N-CH_2), 3.13 (q, $^3J_{HH}$ = 3.0 Hz, $^4J_{HH}$ = 1.4 Hz, 2H, CH_2).

*Synthesis of 1-benzyl-1,4-dihydropyridine-3-carboxylic acid (**1d**).*

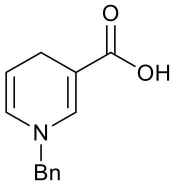

The compound **1d** was synthesised in accordance with previously outlined procedures.[2] Nicotinic acid (2.46 g, 20 mmol) was dissolved in a minimal amount of ethanol and diluted with acetonitrile (40 mL). Benzyl bromide (2.4 mL, 20 mmol) was then added and stirred under reflux at 80 °C for 12 h. The solution was cooled and diethyl ether (50 mL) was added to precipitate the crude product. After filtering off the supernatant and washing the solid with diethyl ether (3 x 10 mL), the bromide salt was obtained as a white powder (3.5 g, 11.9 mmol, 60%). A suspension of 1-benzyl-3-carboxy pyridinium bromide (2.9 g, 10 mmol) in water (200 mL) and dichloromethane (100 mL) was cooled to 0 °C and stirred under a nitrogen atmosphere. Sodium carbonate (6.4 g, 60 mmol) was added in small portions over a period of 10 minutes. Sodium dithionite (7 g, 40 mmol) was then added slowly over a period of 15 minutes. Stirring was continued over a period of 1 hour under a nitrogen stream at 0 °C. The organic layer was washed with water (3 x 100 mL), dried over Na_2SO_4 and the volatiles evaporated under vacuum to afford the yellow product (0.55 g, 2.6 mmol, 26%). Analytical data agrees with previously reported values.[2] **^{1}H NMR** (400 MHz, $CDCl_3$, 298 K) δ/ppm: 7.36–7.30 (m, 3H, Ar-H), 7.25–7.23 (m, 3H, Ar-H), 7.22 (d, $^3J_{HH}$ = 1.4 Hz, 1H, NCH), 5.68 (dq, $^3J_{HH}$ = 8.1 Hz, $^4J_{HH}$ = 1.7 Hz, 1H, NCH), 4.82 (dt, $^3J_{HH}$ = 8.1 Hz, $^4J_{HH}$ = 3.5 Hz, 1H, =CH), 4.29 (s, 2H, N-CH_2), 3.12 (d, $^3J_{HH}$ = 3.2 Hz, 2H, CH_2). **^{13}C NMR** (101 MHz, $CDCl_3$, 298 K), δ/ppm: 173.6 (s), 143.6 (s), 137.1 (s), 129.0 (s), 128.2 (s), 128.0 (s), 127.3 (s), 106.1 (s), 96.7 (s), 57.6 (s), 21.9 (s).

© The Authors under the terms of the Creative Commons Attribution License http://creativecommons.org/licenses/by/3.0/, which permits unrestricted use, provided the original author and source are credited.

3. Synthesis of products.

*Synthesis of 1-benzylpyridin-1-ium tris(pentafluorophenyl)hydroborate (**2**)*

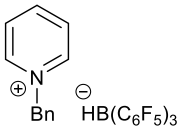

Compound **1a** (17 mg, 0.1 mmol, 1 equiv.) was dissolved in $CDCl_3$ (0.5 mL, 0.2 M) to give a dark red solution to which $B(C_6F_5)_3$ (51 mg, 0.1 mmol, 1 equiv.) was added. This was transferred to an NMR tube to monitor reaction progress. After 30 minutes the reaction showed almost complete conversion to the hydridoborate product **2,** as observed by *in situ* NMR spectroscopy, at this point removal of solvents *in vacuo* gave a dark red viscous oil. Yield: 63 mg, 92 μmol, 91%. **^{1}H NMR** (400 MHz, $CDCl_3$, 298 K) δ/ppm: 8.64 (t, $^{3}J_{HH}$ = 5.8 Hz, 2H, *o*-H), 8.43 (t, $^{3}J_{HH}$ = 7.8 Hz, 1H, *p*-H), 7.97 (t, $^{3}J_{HH}$ = 7.1 Hz, 2H, *m*-H), 7.46–7.39 (m, 3H, Ar-H), 7.27 (d, $^{3}J_{HH}$ = 7.1 Hz, 2H, Ar-H), 5.67 (s, 2H, N-CH_2). Note: B-H not observed. **^{13}C NMR** (101 MHz, $CDCl_3$, 298 K), δ/ppm: 148.2 (dm, $^{1}J_{CF}$ = 240 Hz), 146.2 (s), 143.7 (s), 136.7 (dm, $^{1}J_{CF}$ = 250 Hz), 131.2 (s), 130.4 (s), 130.3 (s), 129.2 (s), 128.9 (s), 66.0 (s). **^{11}B NMR** (128 MHZ, $CDCl_3$, 298 K) δ/ppm: -25.3 (d, $^{1}J_{BH}$ = 66 Hz). **^{19}F NMR** (376 MHz, $CDCl_3$, 298 K) δ/ppm: -133.82 (br. s, 6F, *o*-F), -162.92 (br. s, 3F, *p*-F), -166.34 (br.s, 6F, *m*-F). **IR** ν_{max} (cm^{-1}): 3091 (w), 2378 (w), 2156 (w), 1641 (m), 1570 (w), 1508 (s), 1487 (w), 1451 (s), 1373 (w), 1273 (m), 1213 (w), 1089 (s), 1028 (w), 974 (s), 910 (m), 787 (w), 760 (m), 743 (m), 700 (m), 679 (m), 617 (w), 601 (w), 567 (w). **HRMS** (ES^-) $[M]^-$ $[C_{18}F_{15}BH]^-$ *m/z* calculated: 511.9968, found 511.9984, (ES^+) $[M]^+$ $[C_{12}H_{12}N_1]^+$ *m/z* calculated: 170.0970, found: 170.0963.

*Synthesis of 1-benzyl-3-carbamoylpyridin-1-ium tris(pentafluorophenyl)borate (**3**)*

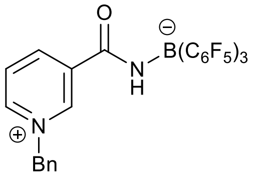

Compound **1b** (42 mg, 0.2 mmol, 1 equiv) was dissolved in $CDCl_3$ (0.5 mL, 0.2 M) to which $B(C_6F_5)_3$ (102 mg, 0.2 mmol, 1 equiv.) was added, yielding an orange solution. This was transferred to a J-Youngs NMR tube and heated for 12 h at 70 °C. A white crystalline solid precipitated out of solution which was isolated and measured by X-ray diffractometry. The mother liquor was retained for isolation of **4** (see below). The remaining solid was washed with cold CH_2Cl_2 (2 x 2 mL) and dried *in vacuo* to garner a white powdery solid. Yield: 63 mg, 87.0 μmol, 44%. m.p. 124–130 °C. **^{1}H NMR** (500 MHz, DMSO-d_6, 298 K) δ/ppm: 9.48 (s, 1H, Ar-H), 9.15 (d, $^{3}J_{HH}$ = 4.9 Hz, 1H, Ar-H), 8.98 (d, $^{3}J_{HH}$ = 7.5 Hz, 1H, Ar-H), 8.19 (t, $^{3}J_{HH}$ = 6.5 Hz, 1H, Ar-H), 7.71 (s, 1H, Ar-H), 7.54 (d, $^{3}J_{HH}$ = 5.3 Hz, 2H, Ph-H), 7.43 (d, $^{3}J_{HH}$ = 6.1 Hz, 3H, Ph-H), 5.85 (s, 2H, CH_2), 5.74 (s, 1H, NH). **^{13}C NMR** (126 MHz, DMSO-d_6, 298 K) δ/ppm: -162.9 (s), 147.8 (dm, $^{1}J_{CF}$ = 240 Hz), 145.2 (s), 144.5 (s), 143.4 (s), 137.6 (dm, $^{1}J_{CF}$ = 240 Hz), 136.9 (s), 135.6 (dm, $^{1}J_{CF}$ = 240 Hz), 134.1 (s), 129.4 (s), 129.3 (s), 129.0 (s), 127.9 (s), 63.4 (s), 54.9 (s). **^{11}B NMR** (160 MHZ, DMSO-d_6, 298 K) δ/ppm: -11.5 (s). **^{19}F NMR** (471 MHz, DMSO-d_6, 298 K) δ/ppm: -133.31 (d, $^{3}J_{FF}$ = 22.4 Hz, 6F, *o*-F), -161.93 (d, $^{3}J_{FF}$ = 21.4 Hz, 3F, *p*-F), -166.23 (t, $^{3}J_{FF}$ = 20.8 Hz, 6F, *m*-F). **IR** ν_{max} (cm^{-1}): 3454 (w), 3071 (w), 1665 (m), 1645 (m), 1625 (w), 1512 (m), 1487 (m), 1458 (s), 1379 (w), 1277 (m), 1180 (w), 1082 (s), 1030 (w), 974 (s), 947 (s), 887 (w), 813 (w), 771

© The Authors under the terms of the Creative Commons Attribution License http://creativecommons.org/licenses/by/3.0/, which permits unrestricted use, provided the original author and source are credited.

(m), 758 (m), 739 (m), 704 (m), 675 (m), 623 (w), 574 (m), 548 (w). **HRMS** (ES^-) $[M–H]^-$ $[C_{31}H_{11}N_2OBF_{15}]^-$ *m/z* calculated: 722.0761, found: 722.0769.

*Synthesis of 1-benzyl-1,4,5,6-tetrahydropyridine-3-carboxamide tris(pentafluorophenyl)borane adduct (**4**).*

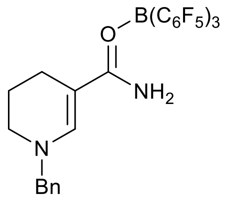

The mother liquor from the formation of **3** was retained with the solvents being subsequently removed under reduced pressure. The yellow/green solid was then washed with cold pentane (2 x 2 mL) and dried *in vacuo* to yield a yellow/green powdery solid. Yield: 61 mg, 83.4 µmol, 42%. m.p. 101–110 °C. **^{1}H NMR** (500 MHz, $CDCl_3$, 298 K) δ/ppm: 7.92 (s, 1H, C=CH), 7.39–7.33 (m, 3H, Ph-H), 7.20 (d, $^3J_{HH}$ = 7.2 Hz, 2H, Ph-H), 5.52 (br. s, 2H, NH_2), 4.42 (s, 2H, CH_2), 3.15 (t, $^3J_{HH}$ = 4.9 Hz, 2H, CH_2), 2.11 (t, $^3J_{HH}$ = 5.7 Hz, 2H, CH_2), 1.91 (br. pent., $^3J_{HH}$ ≈ 5 Hz, 2H, CH_2). **^{13}C NMR** (126 MHz, $CDCl_3$, 298 K) δ/ppm: 169.6 (s), 151.5 (s), 147.9 (dm, $^1J_{CF}$ = 245 Hz), 139.9 (dm, $^1J_{CF}$ = 245 Hz), 137.0 (dm, $^1J_{CF}$ = 245 Hz), 134.5 (s), 129.3 (s), 129.2 (s), 128.8 (s), 128.4 (s), 128.0 (s), 125.5 (s), 90.5 (s), 61.3 (s), 45.7 (s), 20.5 (s), 18.9 (s). **^{11}B NMR** (160 MHz, $CDCl_3$, 298 K) δ/ppm: -2.8 (br. s). **^{19}F NMR** (471 MHz, $CDCL_3$, 298 K) δ/ppm: -133.92 (d, $^3J_{FF}$ = 21.0 Hz, 6F, *o*-F), -158.14 (t, $^3J_{FF}$ = 20.2 Hz, 3F, *p*-F), -163.93 (t, $^3J_{FF}$ = 19.0 Hz, 6F, *m*-F). **IR** ν_{max} (cm^{-1}): 3524 (w), 3420 (w), 1643 (m), 1618 (m), 1558 (w), 1510 (s), 1458 (s), 1418 (m), 1354 (m), 1319 (w), 1279 (m), 1211 (m), 1089 (s), 1028 (w), 976 (s), 877 (m), 806 (m), 773 (m), 764 (m), 750 (m), 734 (m), 696 (m), 675 (m), 625 (w), 609 (w), 574 (w), 556 (w). **HRMS** (ES^+) $[M+H(–B(C_6F_5)_3)]^+$ $[C_{13}H_{17}N_2O]^+$ *m/z* calculated: 217.1341, found: 217.1339.

*Synthesis of 1-benzyl-3-carbamoylpyridin-1-ium-N-d_2-tris(pentafluorophenyl)borate (**5**).*

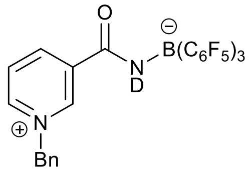

Compound **1c** (44 mg, 0.2 mmol, 1 equiv) was dissolved in $CDCl_3$ (0.5 mL, 0.2 M) to which $B(C_6F_5)_3$ (102 mg, 0.2 mmol, 1 equiv.) was added, yielding an orange solution. This was transferred to a J-Youngs NMR tube and heated for 12 h at 70 °C. A white crystalline solid precipitated out of solution which was isolated and washed with cold pentane (2 x 2 mL) and dried *in vacuo* to garner **5** as a white powdery solid. The mother liquor was retained for isolation of **6** (see below). Yield: 58 mg, 80.0 µmol, 40%. m.p. 119–125 °C. **^{1}H NMR** (500 MHz, DMSO-d_6, 298 K) δ/ppm: 9.47 (br. s, 1H, NCH), 9.15 (s, 1H, NCH), 8.96 (br. s, 1H, =CH), 8.19 (t, $^3J_{HH}$ = 6.8 Hz, 1H, =CH), 7.53 (d, $^3J_{HH}$ = 6.7 Hz, 2H, Ar-H), 7.43 (d, $^3J_{HH}$ = 6.2 Hz, 3H, Ar-H), 5.85 (s, 2H, N-CH_2). **^{13}C NMR** (126 MHz, DMSO-d_6, 298 K) δ/ppm: 162.8 (s), 147.8 (dm, $^1J_{CF}$ = 240 Hz), 145.2 (s), 144.5 (s), 143.4 (s), 137.5 (dm, $^1J_{CF}$ = 240 Hz), 136.9, 135.5 (dm, $^1J_{CF}$ = 250 Hz), 134.0 (s), 129.4 (s), 129.3 (s), 128.9 (s), 127.9 (s), 63.4 (s). **^{11}B NMR** (160 MHz, DMSO-d_6, 298 K) δ/ppm: -11.5 (s). **^{19}F NMR** (471 MHz, DMSO-d_6, 298 K) δ/ppm: -133.31 (d, $^3J_{CF}$ = 22.9 Hz, 6F, *p*-F), -161.9 (t, $^3J_{CF}$ = 21.4 Hz, 3F, *p*-F), -166.3 (t, $^3J_{CF}$ = 20.8 Hz, 6F, *m*-F). **IR** ν_{max} (cm^{-1}): 3464 (w), 2359 (w), 2164 (w), 1665 (m), 1624 (w), 1514 (m), 1487 (m), 1451 (s), 1379 (m),

© The Authors under the terms of the Creative Commons Attribution License http://creativecommons.org/licenses/by/3.0/, which permits unrestricted use, provided the original author and source are credited.

1279 (m), 1199 (w), 1178 (w), 1082 (s), 976 (s), 962 (s), 947 (s), 908 (s), 883 (w), 825 (w), 771 (m), 764 (s), 704 (m), 657 (m).

*Synthesis of 1-benzyl-1,4,5,6-tetrahydropyridine-5-d-3-carboxamide-N,N-d_2 tris(pentafluorophenyl) borane adduct (**6**).*

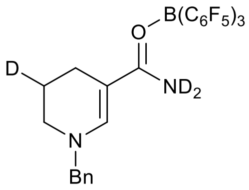

The mother liquor from the formation of **5** was retained with the solvents being subsequently removed under reduced pressure. The orange solid was then washed with cold pentane (2 x 2 mL) and dried *in vacuo* to yield an orange powdery solid. Yield: 56 mg, 76.6 µmol, 38%. m.p. 112–116 °C. **^{1}H NMR** (500 MHz, $CDCl_3$, 298 K) δ/ppm: 7.91 (s, 1H, =CH), 7.40–7.34 (m, 3H, Ar-H), 7.20 (d, $^3J_{HH}$ = 7.0 Hz, 2H, Ar-H), 4.42 (s, 2H, N-CH_2), 3.14 (d, $^3J_{HH}$ = 5.4 Hz, 2H, CH_2), 2.10 (d, $^3J_{HH}$ = 6.1 Hz, 2H, CH_2), 1.91 (pent., $^3J_{HH}$ = 6.1 Hz, 1H, CHD). **^{13}C NMR** (126 MHz, $CDCl_3$, 298 K) δ/ppm: 169.5 (s), 151.5 (s), 147.9 (dm, $^1J_{CF}$ = 240 Hz), 139.8 (dm, $^1J_{CF}$ = 250 Hz), 137.1 (dm, $^1J_{CF}$ = 255 Hz), 134.5 (s), 130.4 (s), 129.3 (s), 128.8 (s), 128.0 (s), 61.3 (s), 45.7 (s), 22.5 (s), 20.5 (s). **^{11}B NMR** (160 MHz, $CDCl_3$, 298 K) δ/ppm: -2.78 (br. s). **^{19}F NMR** (471 MHz, $CDCL_3$, 298 K) δ/ppm: -133.93 (d, $^3J_{FF}$ = 22.1 Hz, 6F, *o*-F), -158.14 (t, $^3J_{FF}$ = 20.4 Hz, 3F, *p*-F), -163.92 (t, $^3J_{FF}$ = 18.7 Hz, 6F, *m*-F). **IR** ν_{max} (cm^{-1}): 3082 (w), 2160 (w), 2035 (w), 1687 (s), 1647 (m), 1516 (m), 1425 (s), 1386 (m), 1342 (m), 1275 (m), 1198 (w), 1182 (m), 1083 (s), 1033 (w), 975 (s), 934 (m), 906 (m), 881 (m), 812 (m),773 (m), 758 (s), 724 (s), 706 (s), 677 (s), 665 (s), 650 (m).

*Synthesis of ((1-benzylpyridin-1-ium-3-carbonyl)oxy)tris(pentafluorophenyl)borate (**7**)*

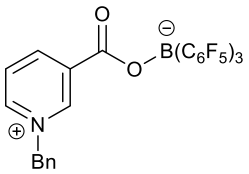

Compound **1d** (44 mg, 0.1 mmol, 1 equiv) was dissolved in $CDCl_3$ (0.5 mL, 0.2 M) to which $B(C_6F_5)_3$ (102 mg, 0.1 mmol, 1 equiv.) was added, yielding an orange solution. This was transferred to a J-Youngs NMR tube and heated for 12 h at 70 °C. An orange precipitate formed which was separated from the supernatant and washed with pentane (2 x 2 mL) then dried *in vacuo* to garner a light orange powdery solid. Yield: 52 mg, 71.7 µmol, 36%. m.p. 137–144 °C. **^{1}H NMR** (500 MHz, DMSO-d_6, 298 K) δ/ppm: 9.5 (s, 1H, NCH), 9.31 (d, $^3J_{HH}$ = 5.9 Hz, 1H, NCH), 8.89 (d, $^3J_{HH}$ = 7.9 Hz, 1H, =CH), 8.27 (t, $^3J_{HH}$ = 7.0 Hz, 1H, =CH), 7.55 (br. s, 2H, Ar-H), 7.41 (br. s, 3H, Ar-H), 5.92 (s, 2H, N-CH_2). **^{13}C NMR** (126 MHz, DMSO-d_6, 298 K) δ/ppm: 147.1 (dm, $^1J_{CF}$ = 245 Hz), 146.8 (s), 145.3 (s), 144.9 (s), 117.4 (dm, $^1J_{CF}$ = 250 Hz), 135.7 (dm, $^1J_{CF}$ = 245 Hz), 133.6 (s), 133.4 (s), 129.4 (s), 129.0 (s), 128.6 (s), 63.5 (s). **^{11}B NMR** (160 MHZ, DMSO-d_6, 298 K) δ/ppm: -4.1 (br. s). **^{19}F NMR** (471 MHz, DMSO-d_6, 298 K) δ/ppm: -134.10 (d, $^3J_{FF}$ = 22.0 Hz, 6F, *o*-F), -160.65 (t, $^3J_{FF}$ = 21.2 Hz, 3F, *p*-F), -165.57 (t, $^3J_{FF}$ = 20.2 Hz, 6F, *m*-F). **IR** ν_{max} (cm^{-1}): 2978 (w), 2872 (w), 1645 (w), 1514 (m), 1456 (s), 1275 (m), 1182 (m), 1082 (s), 976 (s), 906 (m), 808 (m), 772 (w), 758 (w), 731 (m), 706 (m), 675 (m), 623 (m).

© The Authors under the terms of the Creative Commons Attribution License http://creativecommons.org/licenses/by/3.0/, which permits unrestricted use, provided the original author and source are credited.

4. NMR spectra.

4.1 NMR spectra of starting materials.

^{1}H NMR (400 MHz, $CDCl_3$, 298 K) spectrum of 1-benzyl-1,4-dihydropyridine (**1a**).

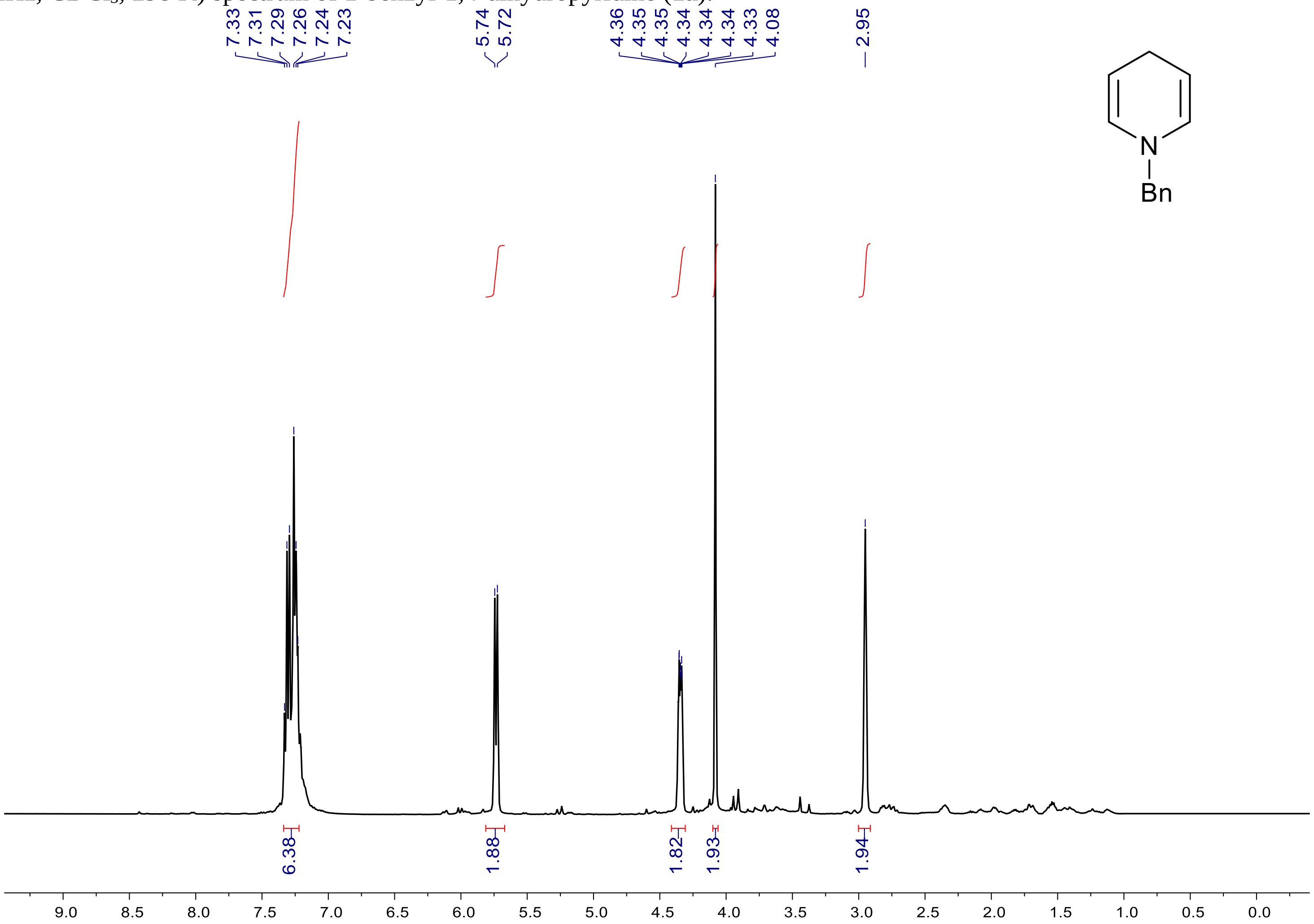

© The Authors under the terms of the Creative Commons Attribution License http://creativecommons.org/licenses/by/3.0/, which permits unrestricted use, provided the original author and source are credited.

^{13}**C NMR** (101 MHz, $CDCl_3$, 298 K) spectrum of 1-benzyl-1,4-dihydropyridine (**1a**).

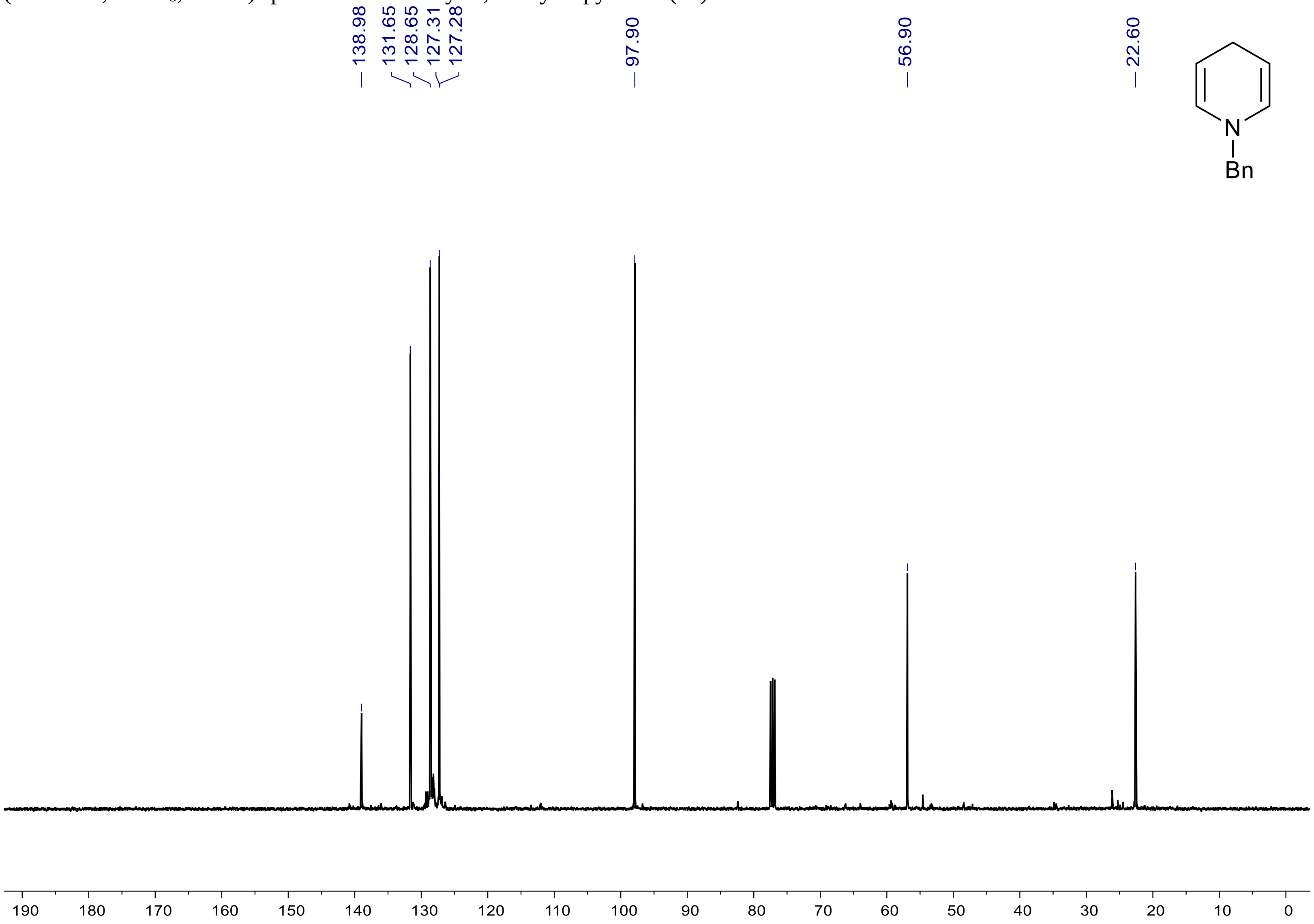

© The Authors under the terms of the Creative Commons Attribution License http://creativecommons.org/licenses/by/3.0/, which permits unrestricted use, provided the original author and source are credited.

Supplementary material for [Author], [Year], [Title], *Phil. Trans. R. Soc. A.* doi: 10.1098/rsta.[paper ID in form xxxx.xxxx e.g. 10.1098/rsta.2014.0049]

[1]**H NMR** (400 MHz, $CDCl_3$, 298 K) spectrum of 1-benzyl-1,4-dihydronicotinamide (**1b**).

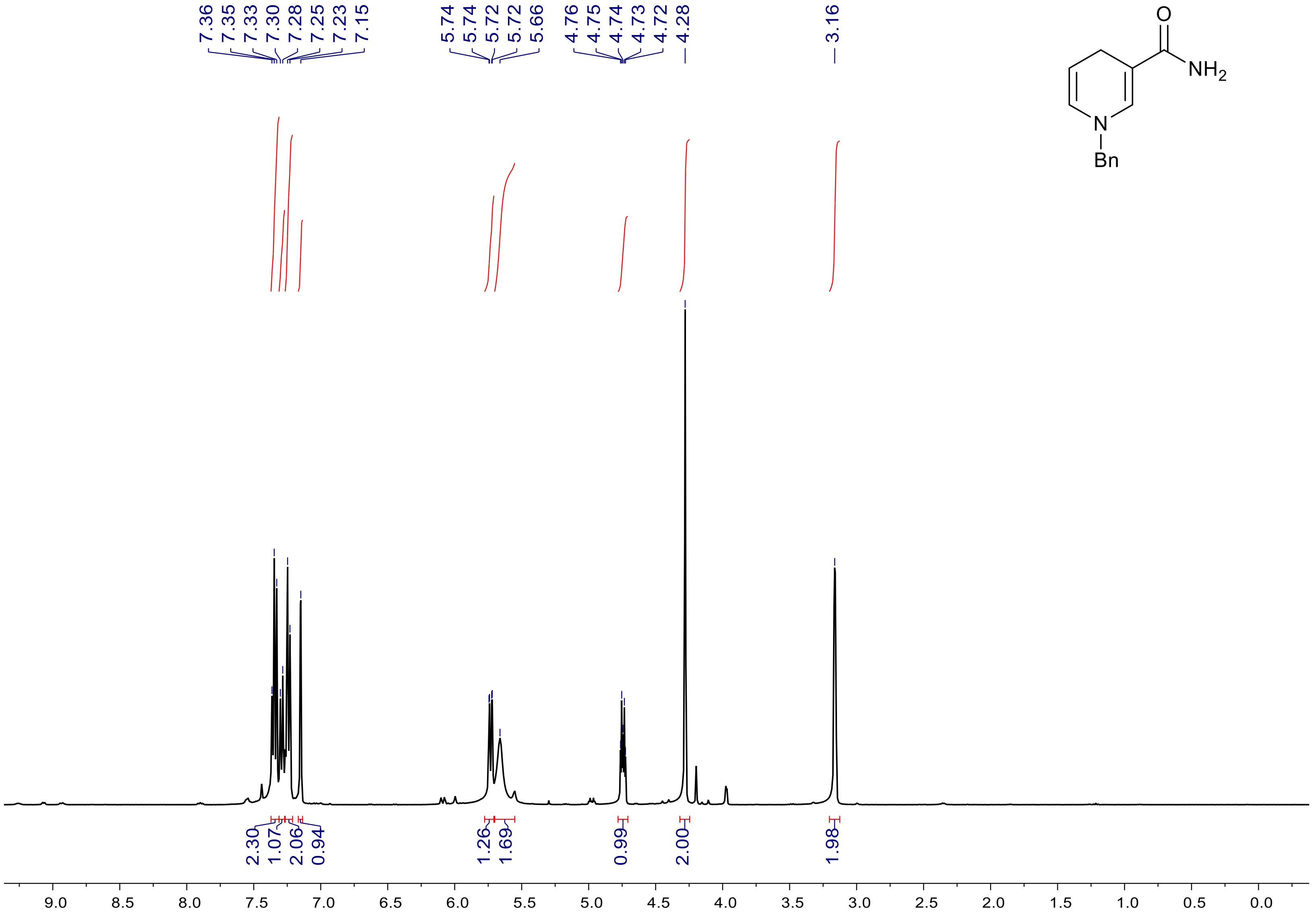

© The Authors under the terms of the Creative Commons Attribution License http://creativecommons.org/licenses/by/3.0/, which permits unrestricted use, provided the original author and source are credited.

^{13}C NMR (101 MHz, $CDCl_3$, 298 K) spectrum of 1-benzyl-1,4-dihydronicotinamide (**1b**).

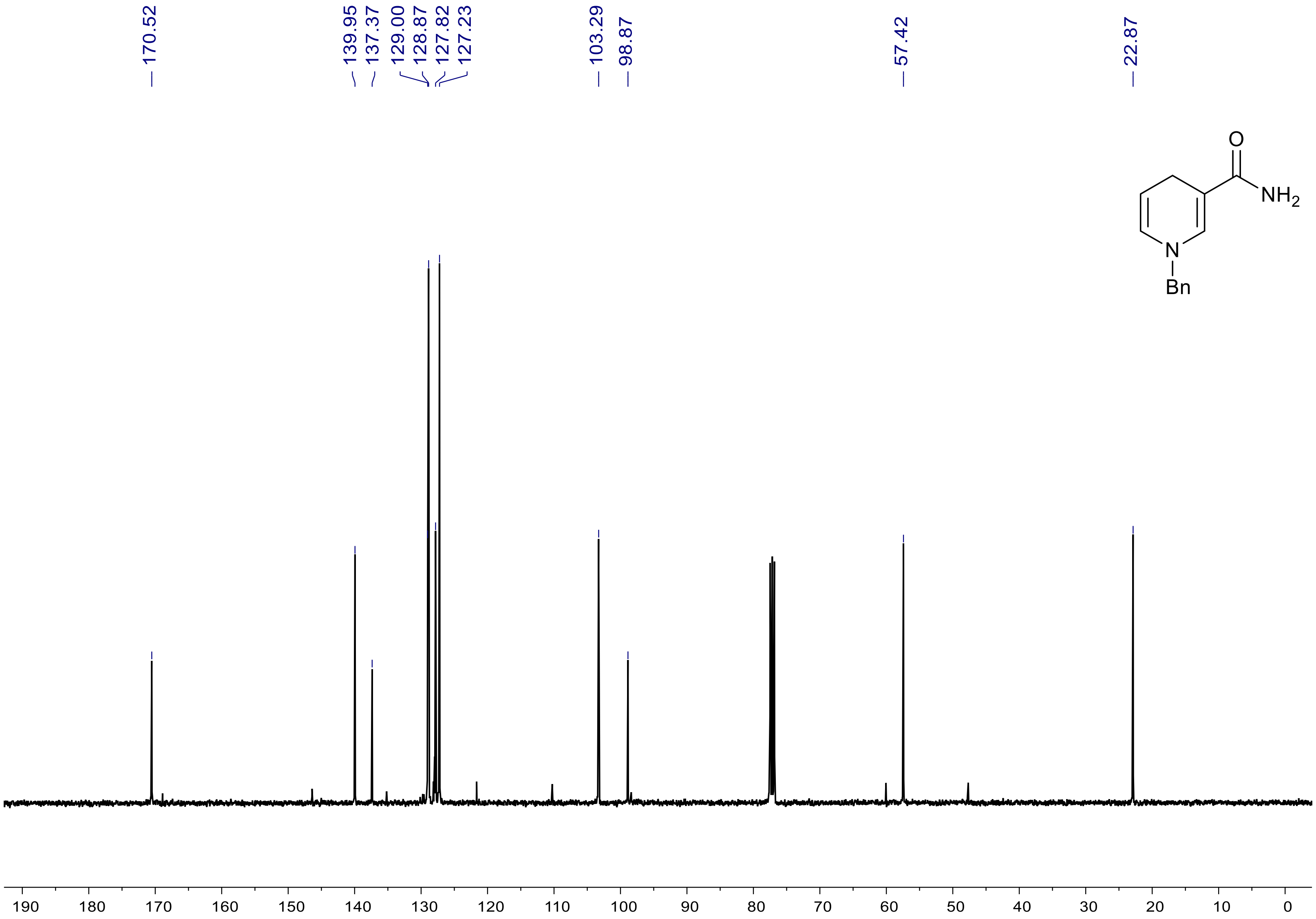

© The Authors under the terms of the Creative Commons Attribution License http://creativecommons.org/licenses/by/3.0/, which permits unrestricted use, provided the original author and source are credited.

Supplementary material for [Author], [Year], [Title], *Phil. Trans. R. Soc. A.* doi: 10.1098/rsta.[paper ID in form xxxx.xxxx e.g. 10.1098/rsta.2014.0049]

^{1}H NMR (400 MHz, $CDCl_3$, 298 K) spectrum of (^{2}H,^{2}H-N)-1-benzyl-1,4-dihydronicotinamide (**1c**).

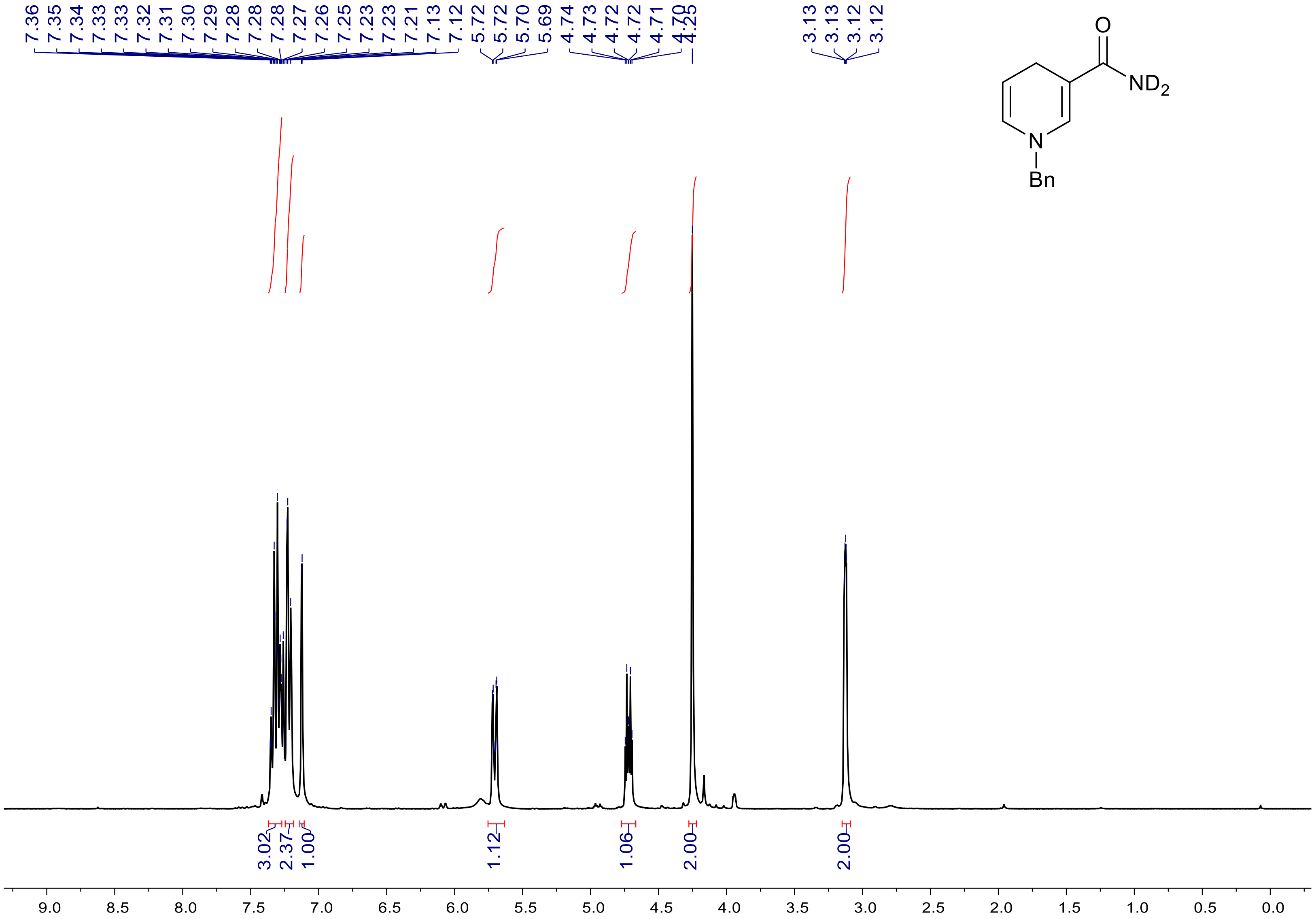

© The Authors under the terms of the Creative Commons Attribution License http://creativecommons.org/licenses/by/3.0/, which permits unrestricted use, provided the original author and source are credited.

^{1}H NMR (400 MHz, $CDCl_3$, 298 K) spectrum of 1-benzyl-1,4-dihydropyridine-3-carboxylic acid (**1d**).

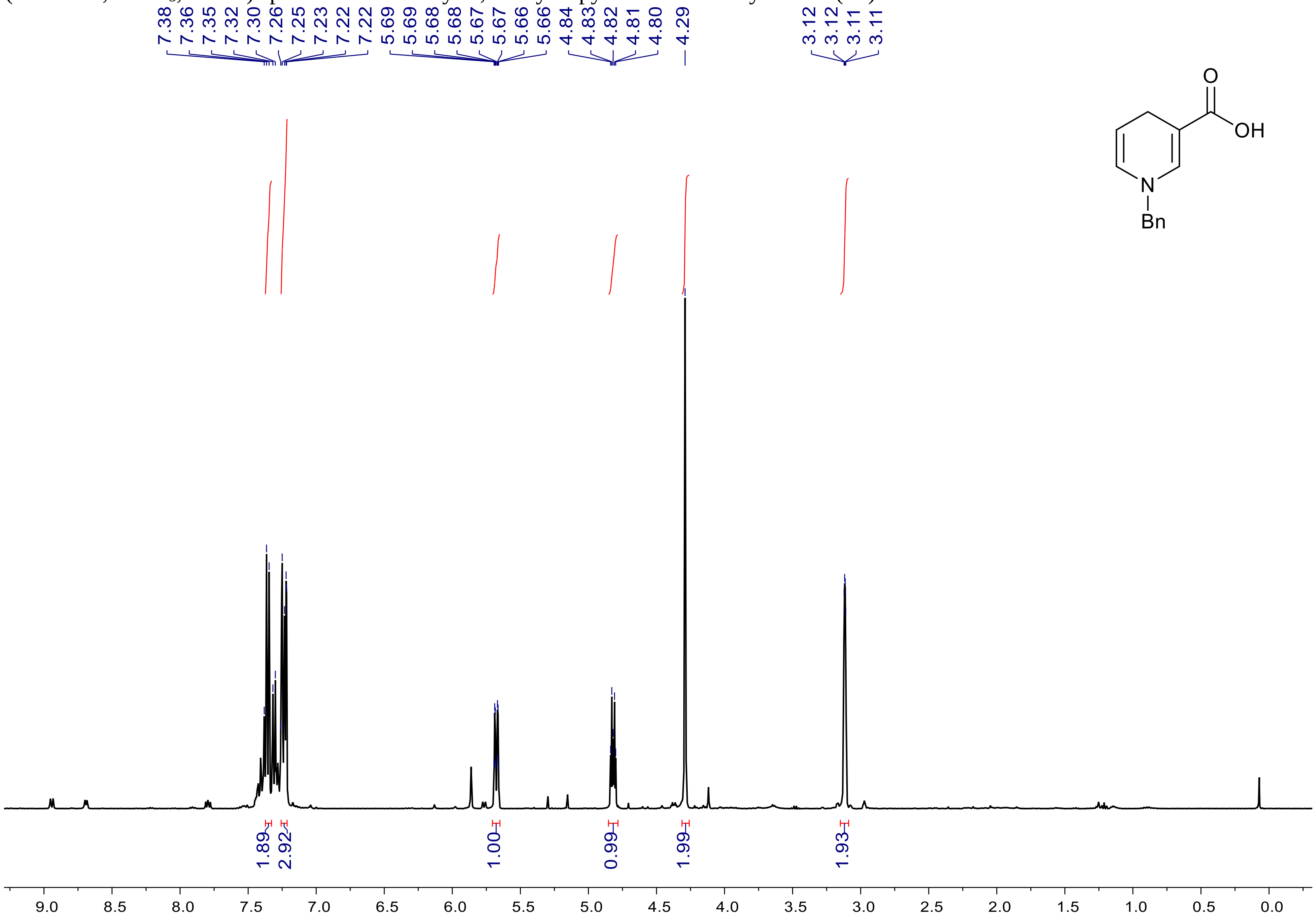

© The Authors under the terms of the Creative Commons Attribution License http://creativecommons.org/licenses/by/3.0/, which permits unrestricted use, provided the original author and source are credited.

^{13}C NMR (101 MHz, $CDCl_3$, 298 K) spectrum of 1-benzyl-1,4-dihydropyridine-3-carboxylic acid (**1d**).

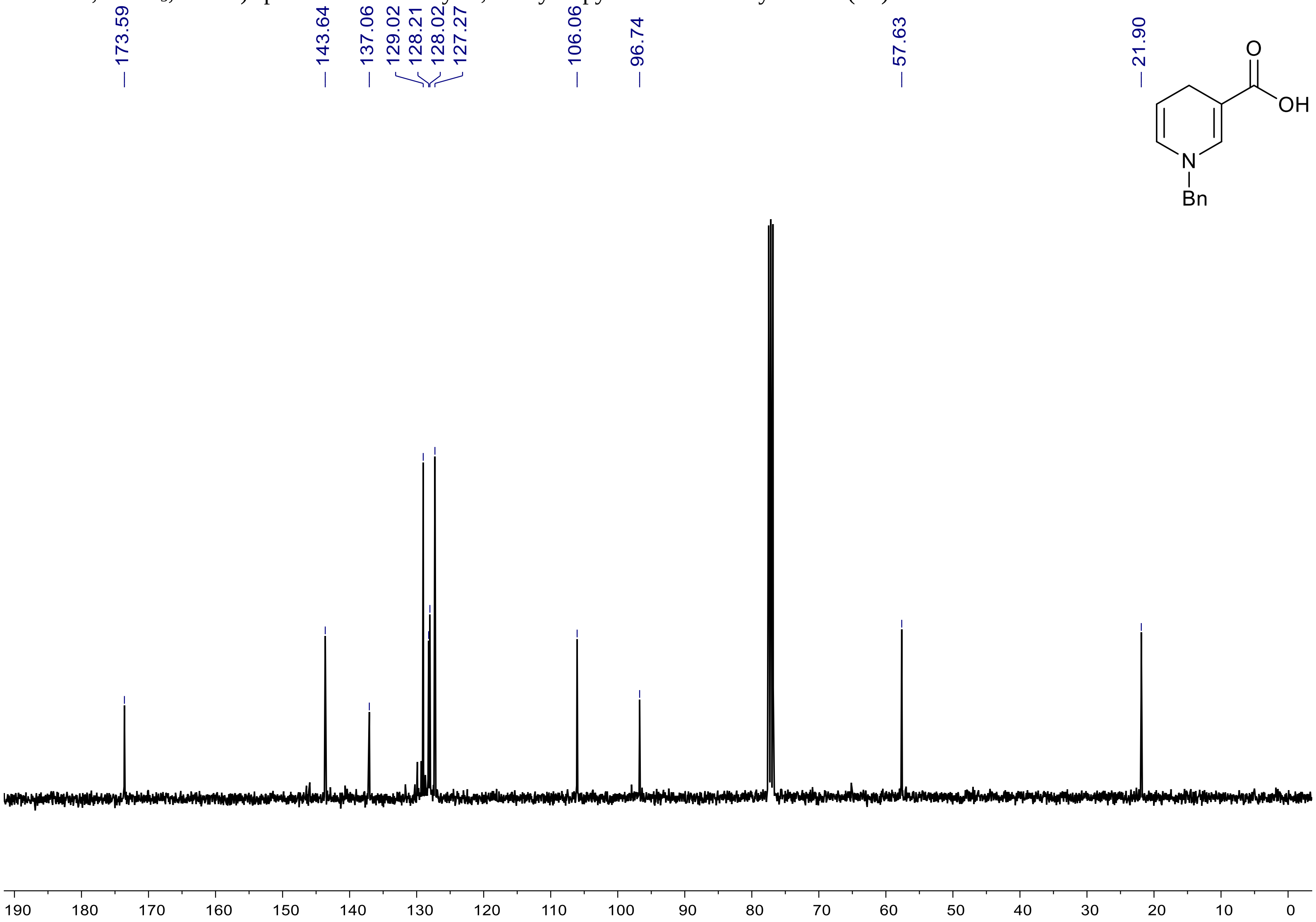

© The Authors under the terms of the Creative Commons Attribution License http://creativecommons.org/licenses/by/3.0/, which permits unrestricted use, provided the original author and source are credited.

4.2 NMR spectra of products.

^{1}**H NMR** (400 MHz, $CDCl_3$, 298 K) spectrum of 1-benzylpyridin-1-ium tris(pentafluorophenyl)hydroborate (**2**).

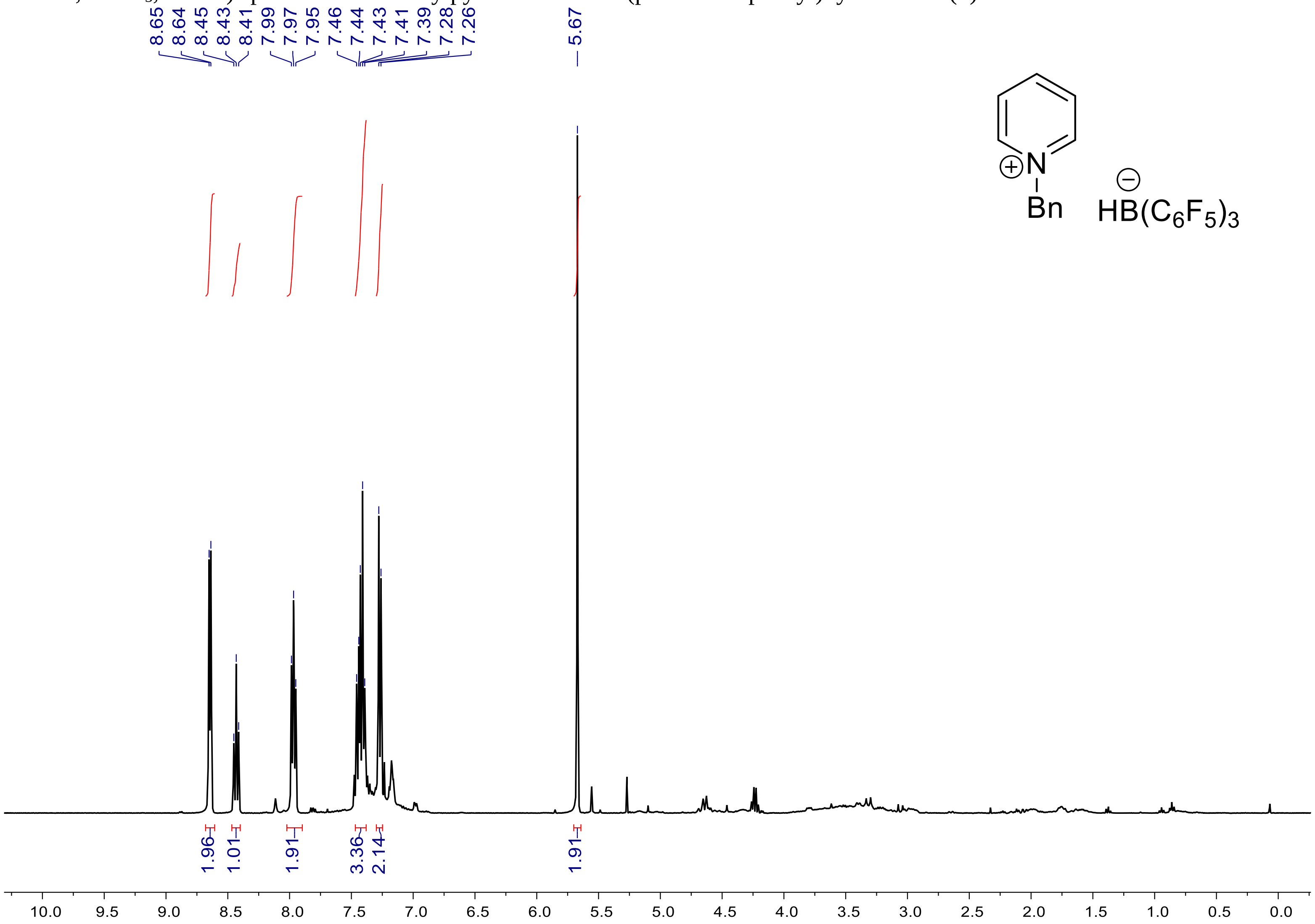

© The Authors under the terms of the Creative Commons Attribution License http://creativecommons.org/licenses/by/3.0/, which permits unrestricted use, provided the original author and source are credited.

^{13}C NMR (101 MHz, $CDCl_3$, 298 K) spectrum of 1-benzylpyridin-1-ium tris(pentafluorophenyl)hydroborate (**2**).

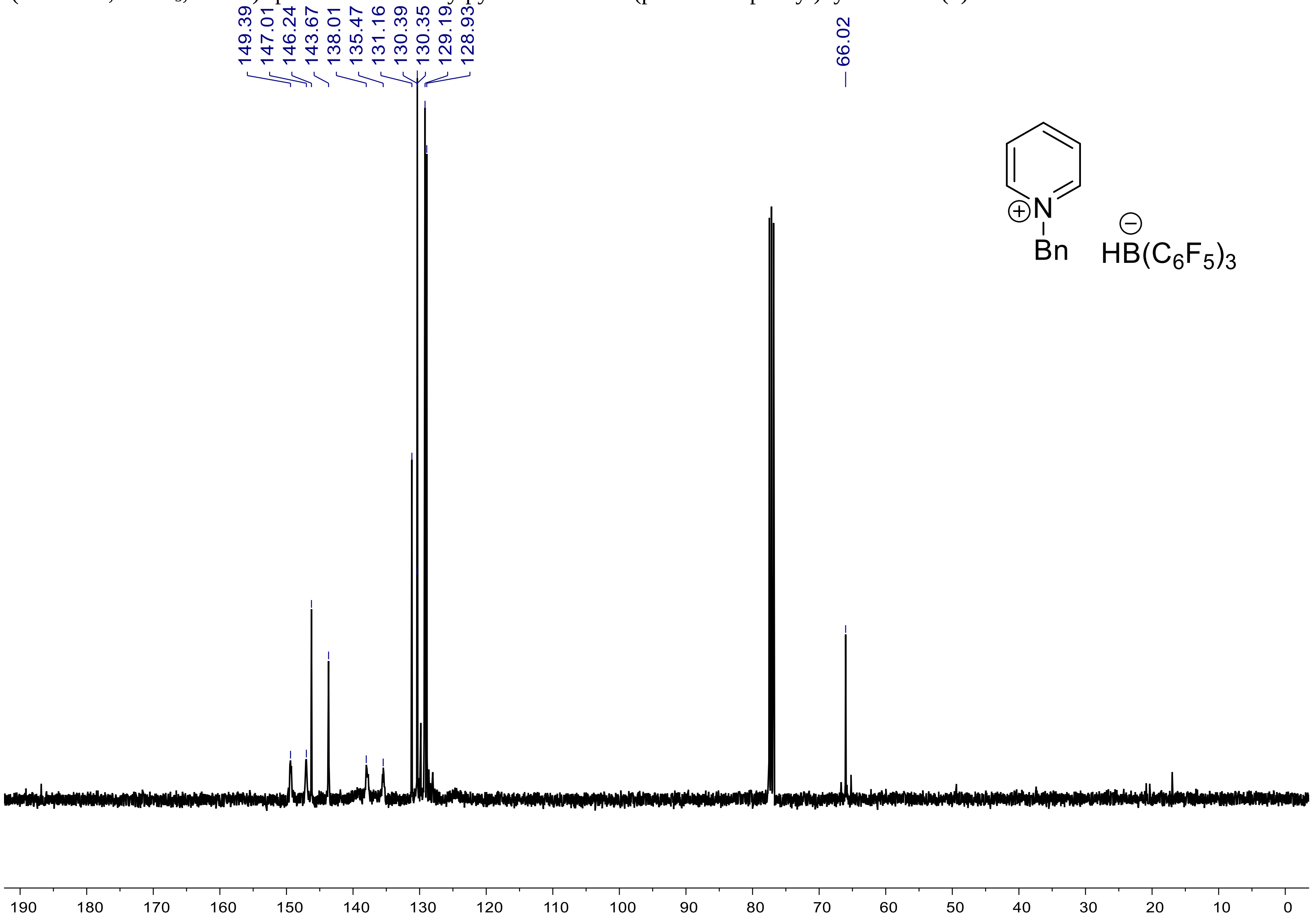

© The Authors under the terms of the Creative Commons Attribution License http://creativecommons.org/licenses/by/3.0/, which permits unrestricted use, provided the original author and source are credited.

^{11}**B NMR** (128 MHz, $CDCl_3$, 298 K) spectrum of 1-benzylpyridin-1-ium tris(pentafluorophenyl)hydroborate (**2**).

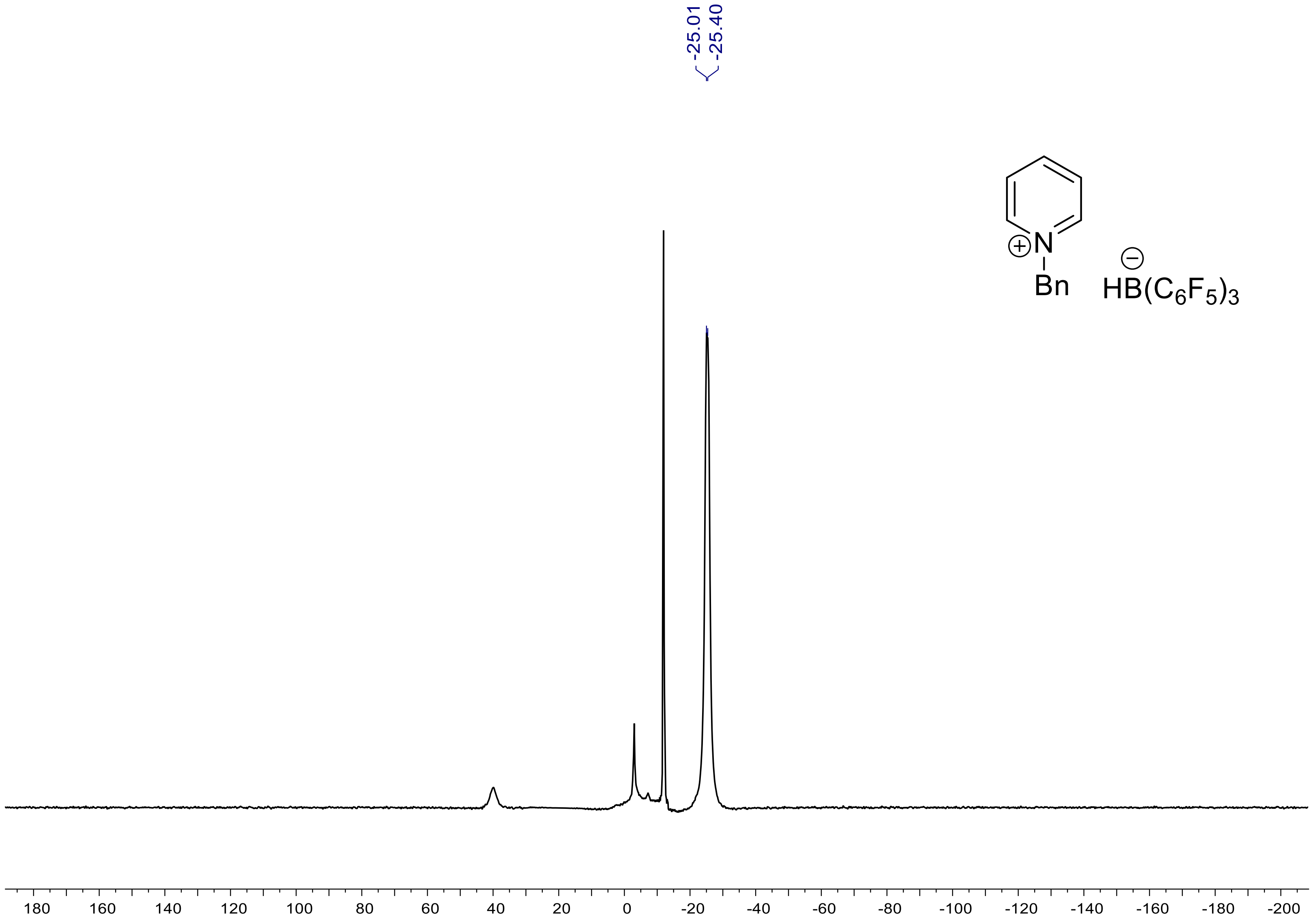

© The Authors under the terms of the Creative Commons Attribution License http://creativecommons.org/licenses/by/3.0/, which permits unrestricted use, provided the original author and source are credited.

^{19}**F NMR** (471 MHz, DMSO-d$_6$, 298 K) δ/ppm: spectrum of 1-benzylpyridin-1-ium tris(pentafluorophenyl)hydroborate (**2**).

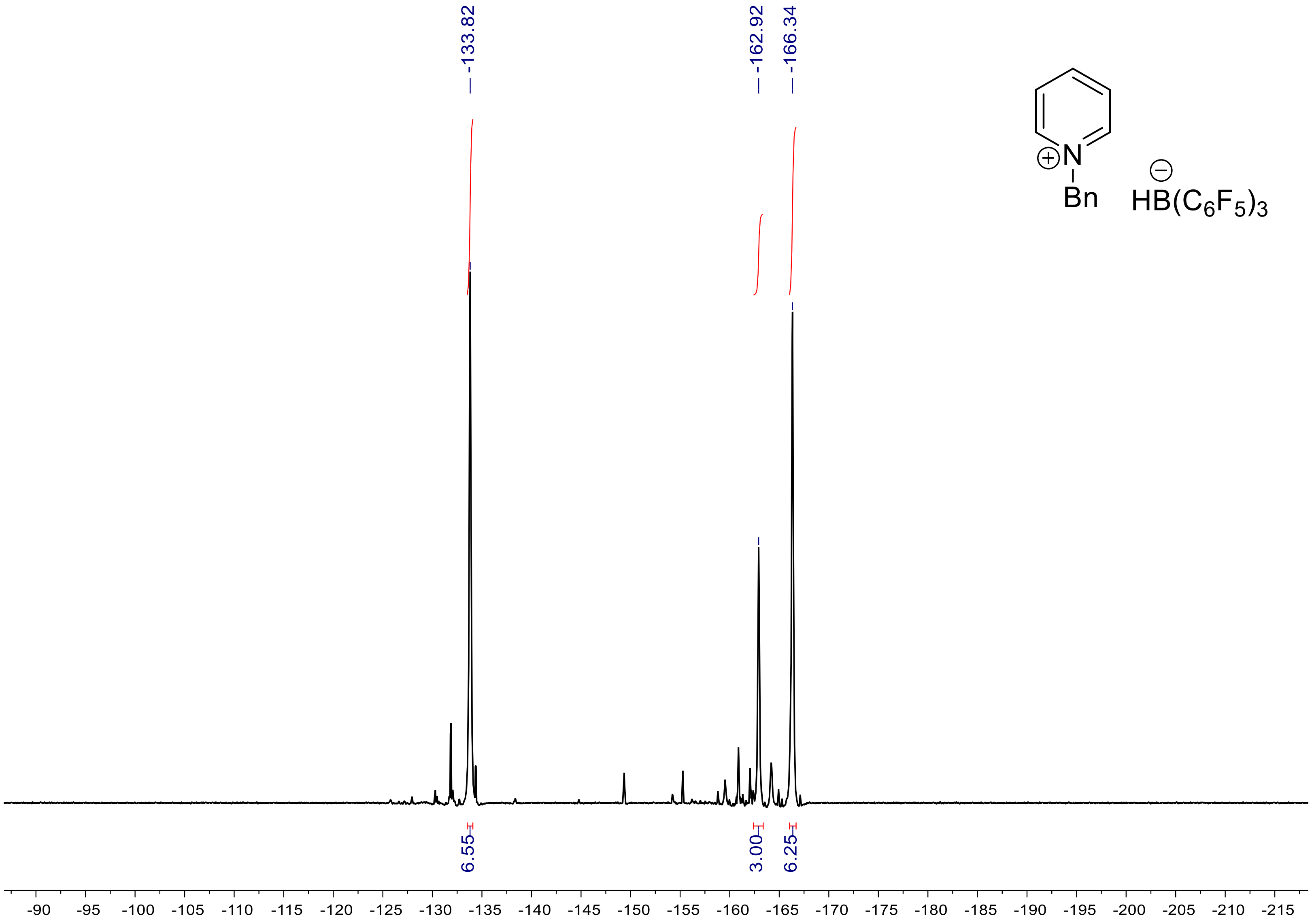

© The Authors under the terms of the Creative Commons Attribution License http://creativecommons.org/licenses/by/3.0/, which permits unrestricted use, provided the original author and source are credited.

^{1}H NMR (500 MHz, DMSO-d_6, 298 K) spectrum of 1-benzyl-3-carbamoylpyridin-1-ium tris(pentafluorophenyl)borate (**3**).

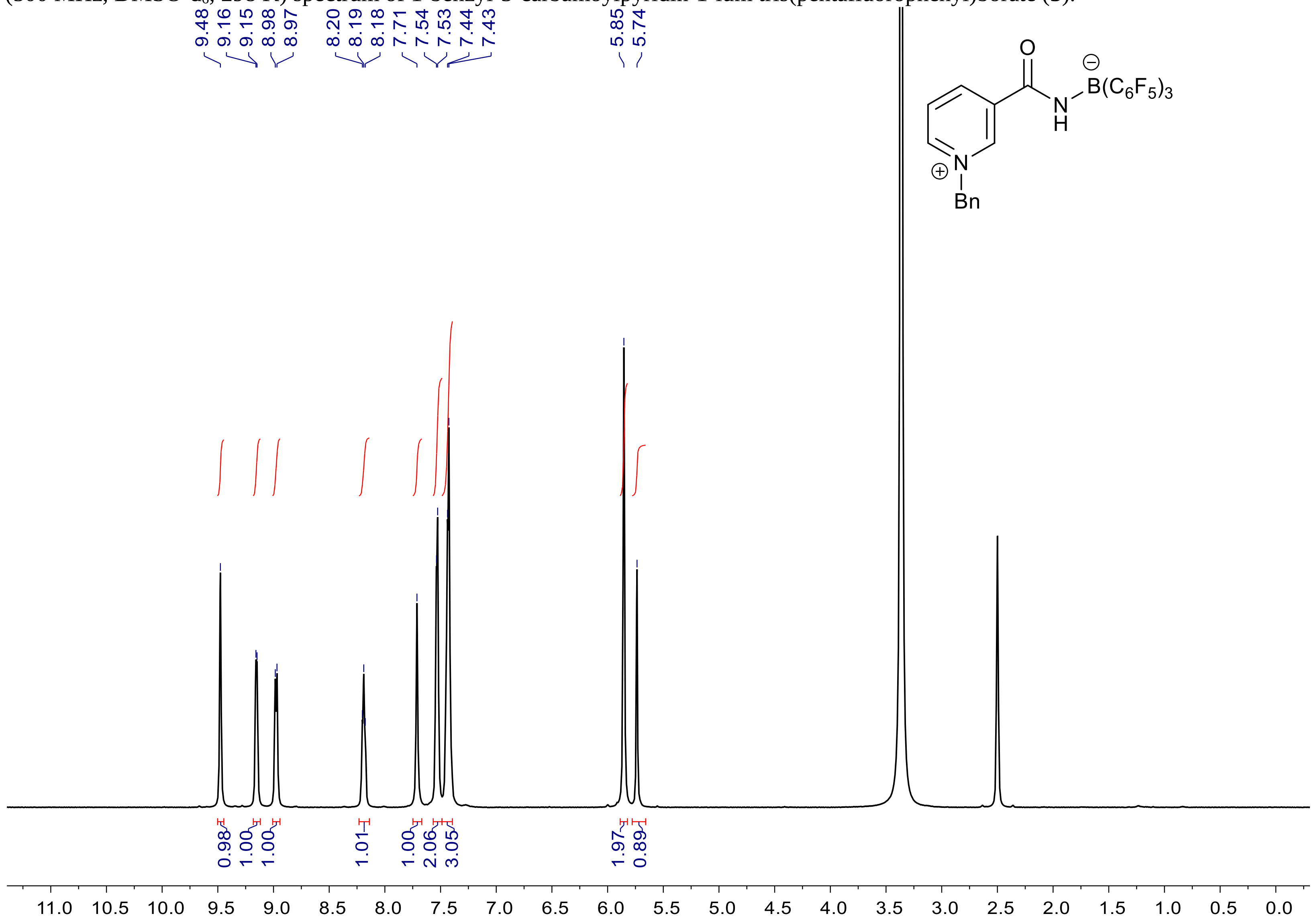

© The Authors under the terms of the Creative Commons Attribution License http://creativecommons.org/licenses/by/3.0/, which permits unrestricted use, provided the original author and source are credited.

^{13}C NMR (126 MHz, DMSO-d$_6$, 298 K) spectrum of 1-benzyl-3-carbamoylpyridin-1-ium tris(pentafluorophenyl)borate (**3**).

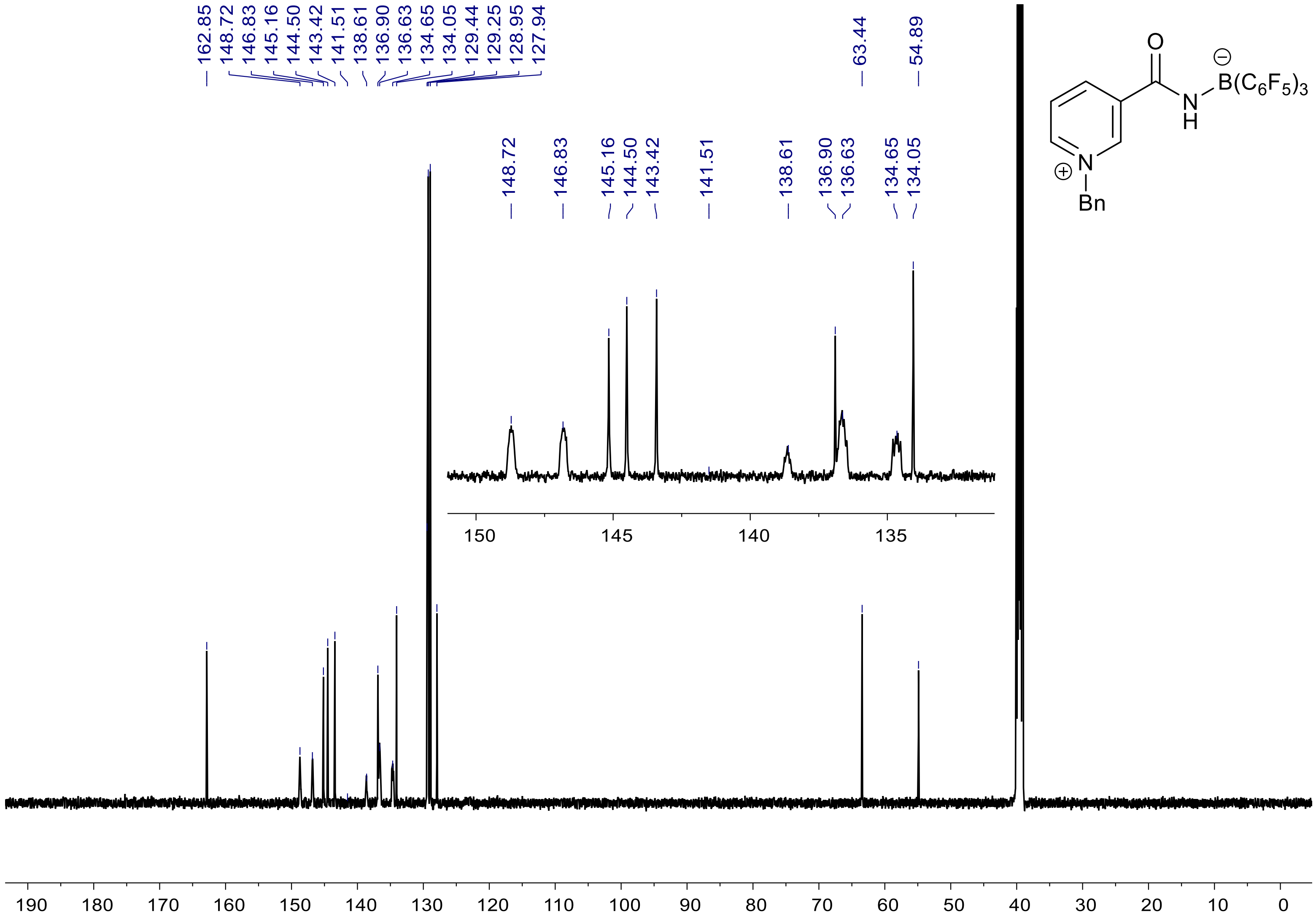

© The Authors under the terms of the Creative Commons Attribution License http://creativecommons.org/licenses/by/3.0/, which permits unrestricted use, provided the original author and source are credited.

^{11}B NMR (160 MHZ, DMSO-d$_6$, 298 K) δ/ppm: spectrum of 1-benzyl-3-carbamoylpyridin-1-ium tris(pentafluorophenyl)borate (**3**).

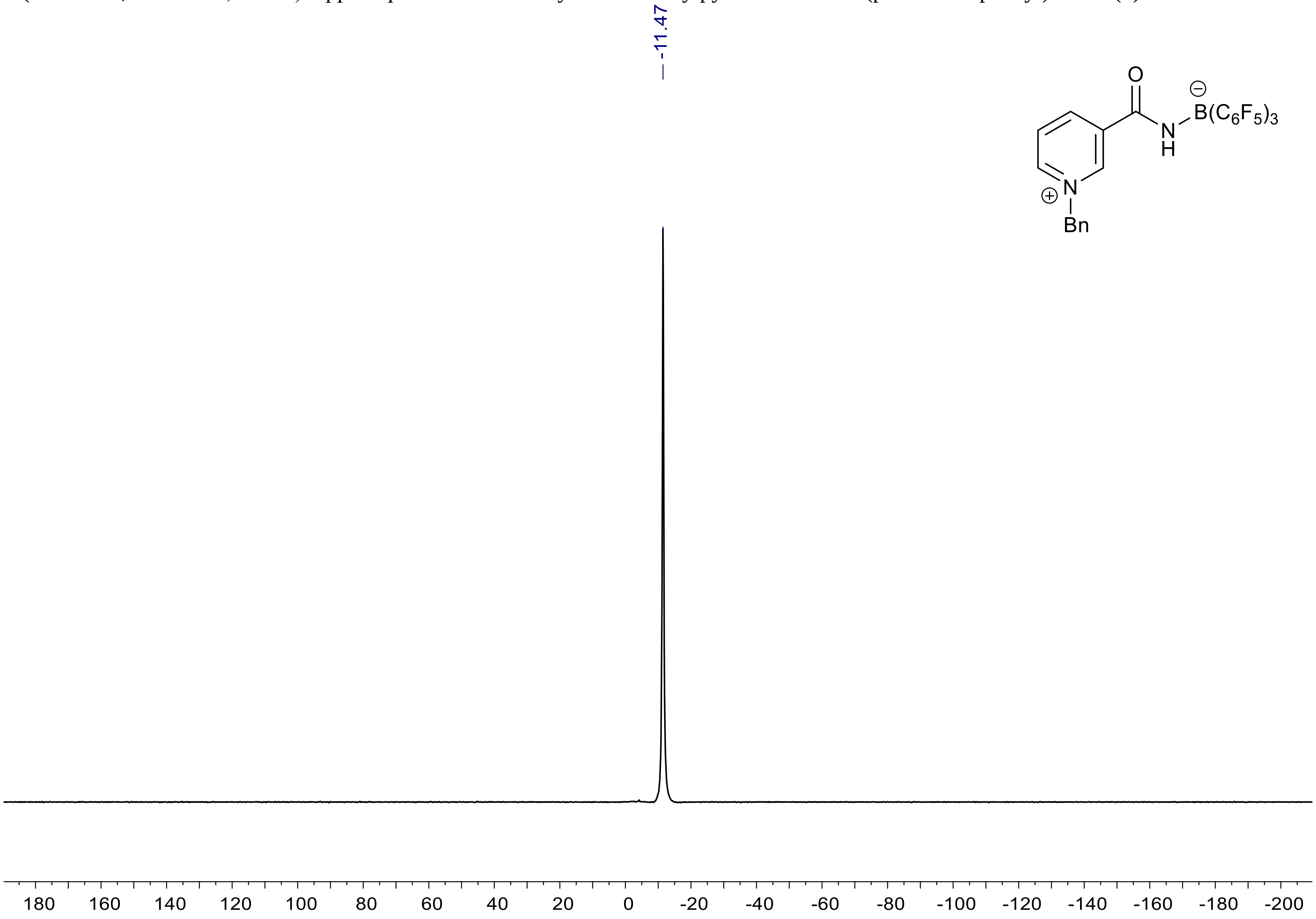

© The Authors under the terms of the Creative Commons Attribution License http://creativecommons.org/licenses/by/3.0/, which permits unrestricted use, provided the original author and source are credited.

^{19}F NMR (471 MHz, DMSO-d_6, 298 K) δ/ppm: spectrum of 1-benzyl-3-carbamoylpyridin-1-ium tris(pentafluorophenyl)borate (**3**).

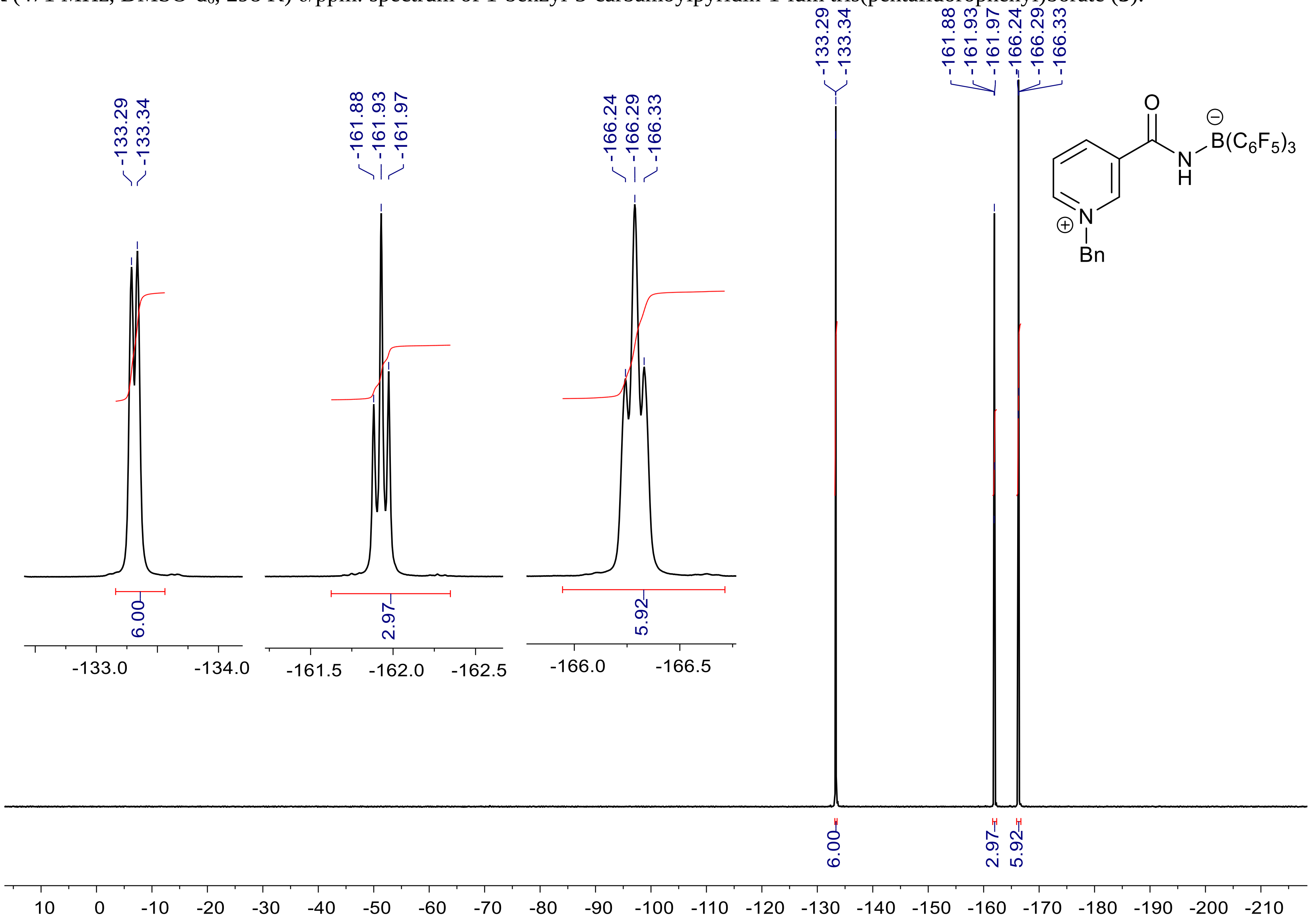

© The Authors under the terms of the Creative Commons Attribution License http://creativecommons.org/licenses/by/3.0/, which permits unrestricted use, provided the original author and source are credited.

¹H NMR (500 MHz, $CDCl_3$, 298 K) spectrum of 1-benzyl-3-carbamoylpyridin-1-ium tris(pentafluorophenyl)borate (**4**).

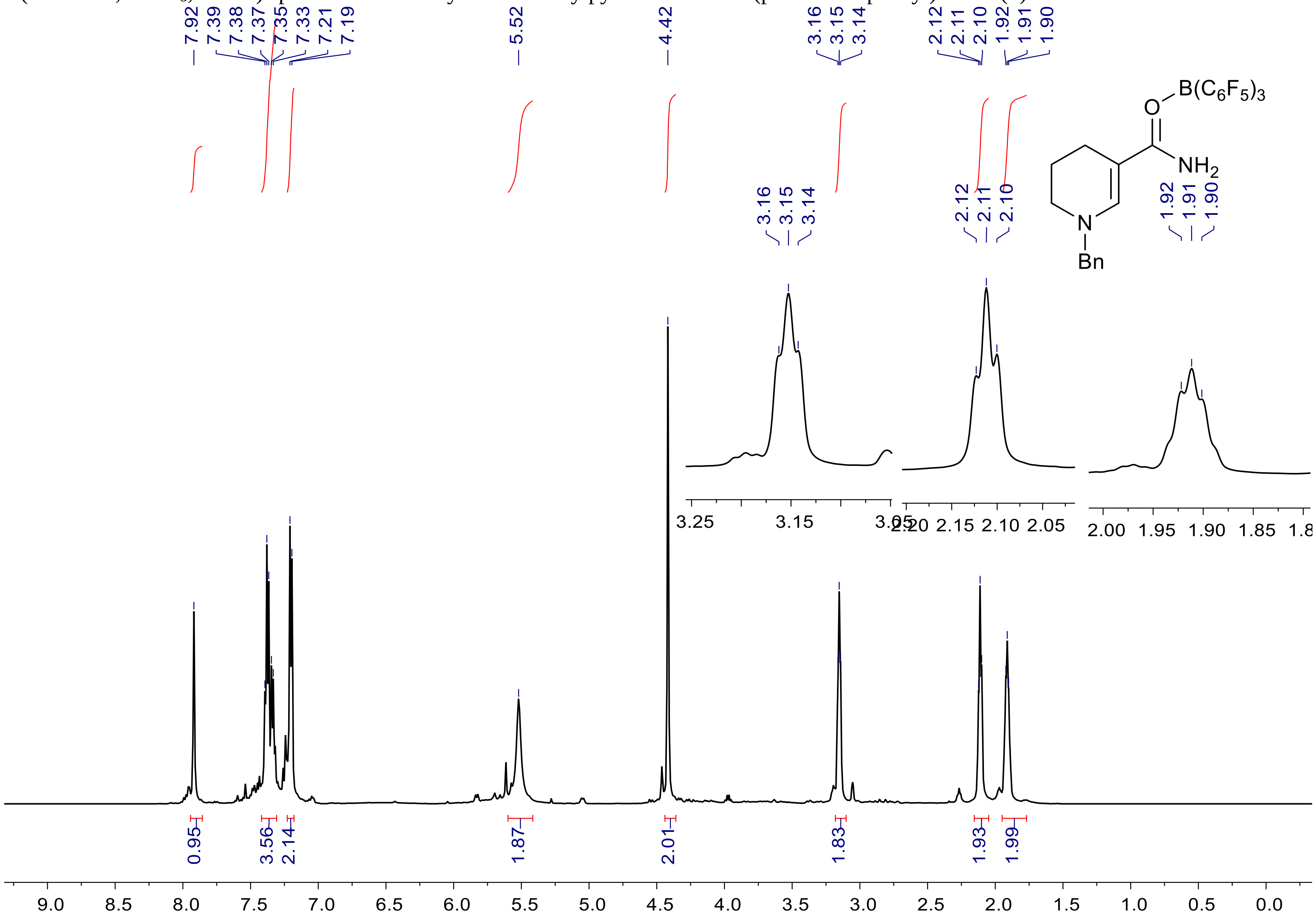

© The Authors under the terms of the Creative Commons Attribution License http://creativecommons.org/licenses/by/3.0/, which permits unrestricted use, provided the original author and source are credited.

^{13}C NMR (126 MHz, $CDCl_3$, 298 K) spectrum of 1-benzyl-3-carbamoylpyridin-1-ium tris(pentafluorophenyl)borate (**4**).

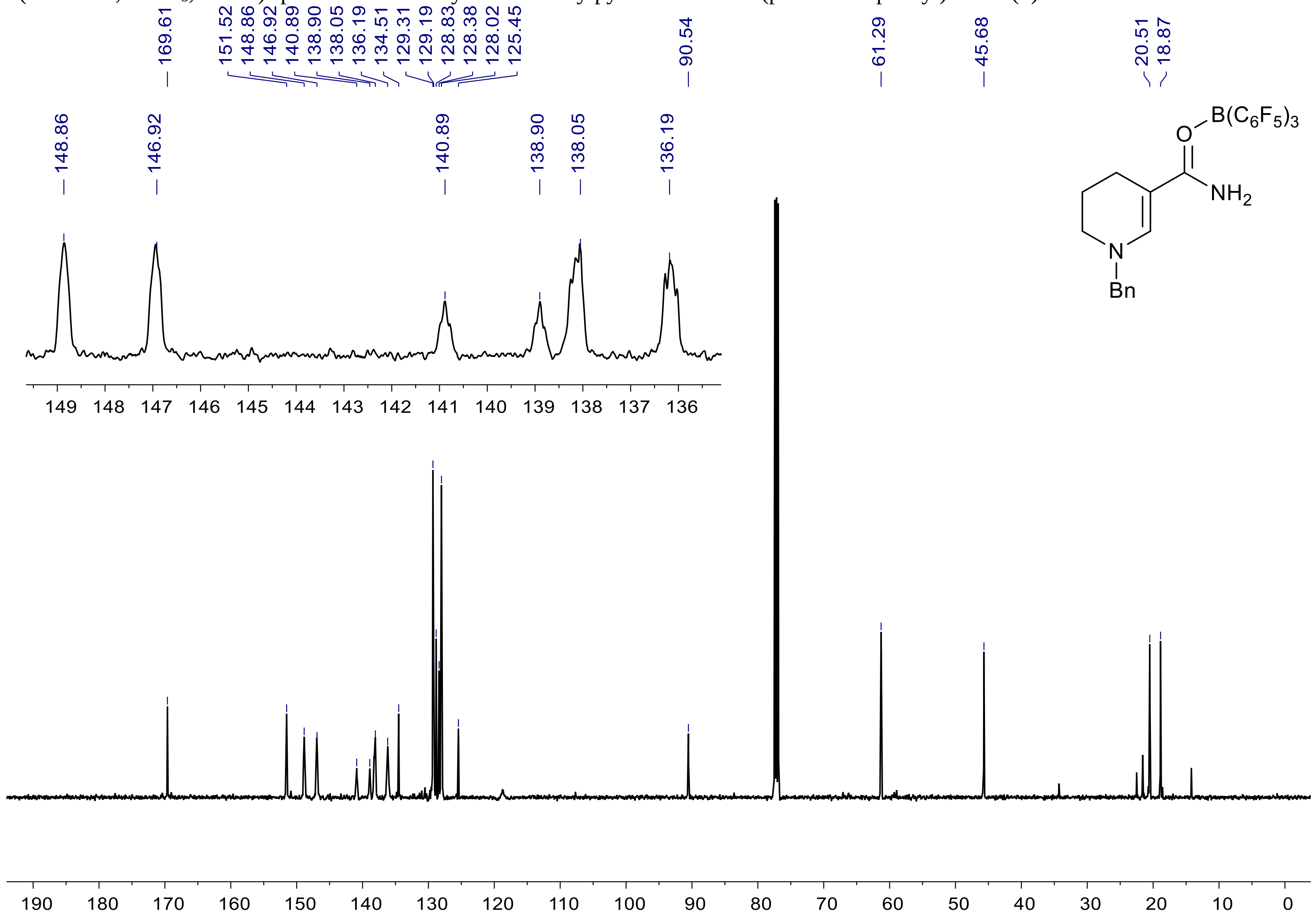

© The Authors under the terms of the Creative Commons Attribution License http://creativecommons.org/licenses/by/3.0/, which permits unrestricted use, provided the original author and source are credited.

^{11}B NMR (160 MHZ, $CDCl_3$, 298 K) δ/ppm: spectrum of 1-benzyl-3-carbamoylpyridin-1-ium tris(pentafluorophenyl)borate (**4**).

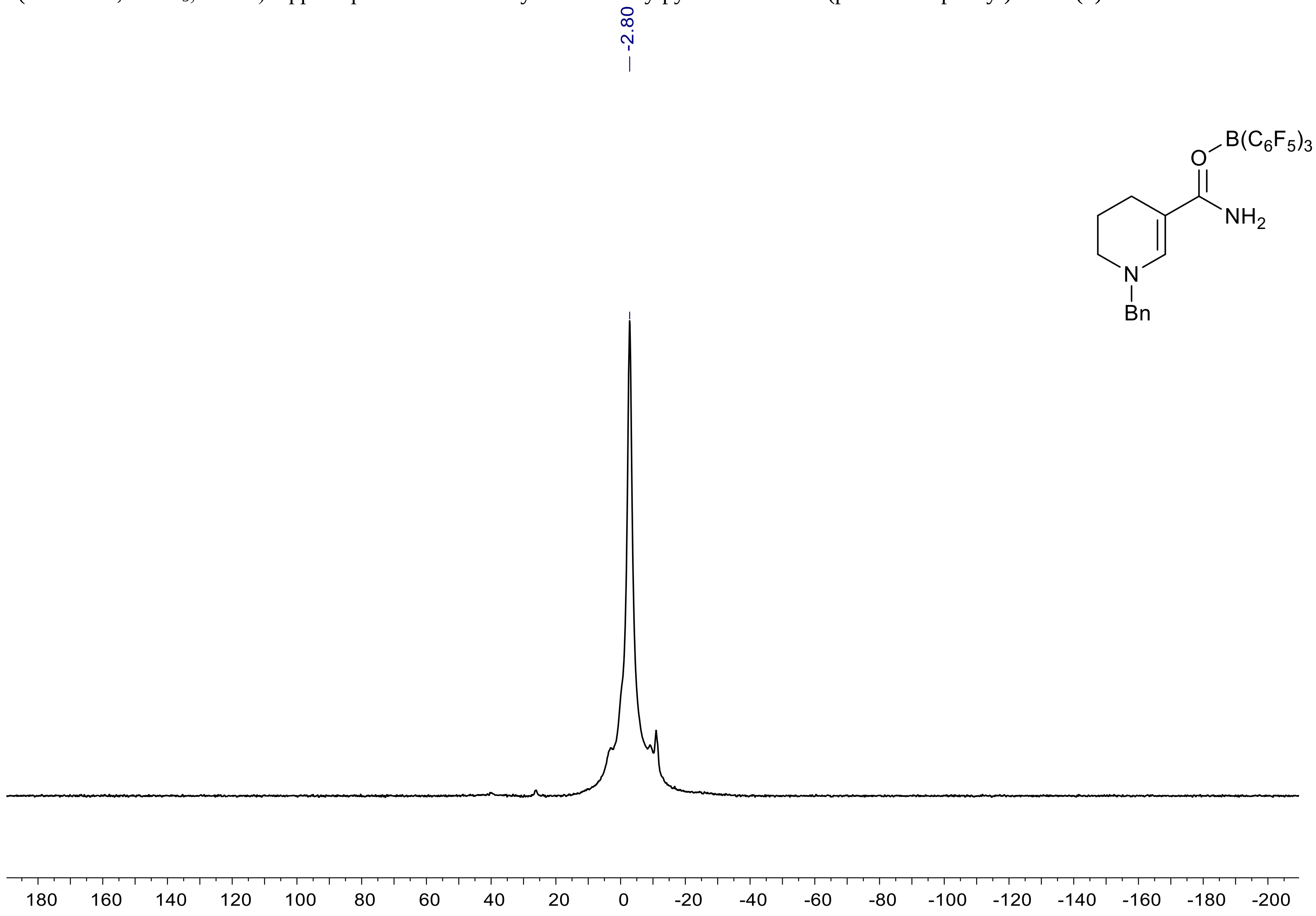

© The Authors under the terms of the Creative Commons Attribution License http://creativecommons.org/licenses/by/3.0/, which permits unrestricted use, provided the original author and source are credited.

^{19}F NMR (471 MHz, $CDCl_3$, 298 K) δ/ppm: spectrum of 1-benzyl-3-carbamoylpyridin-1-ium tris(pentafluorophenyl)borate (**4**).

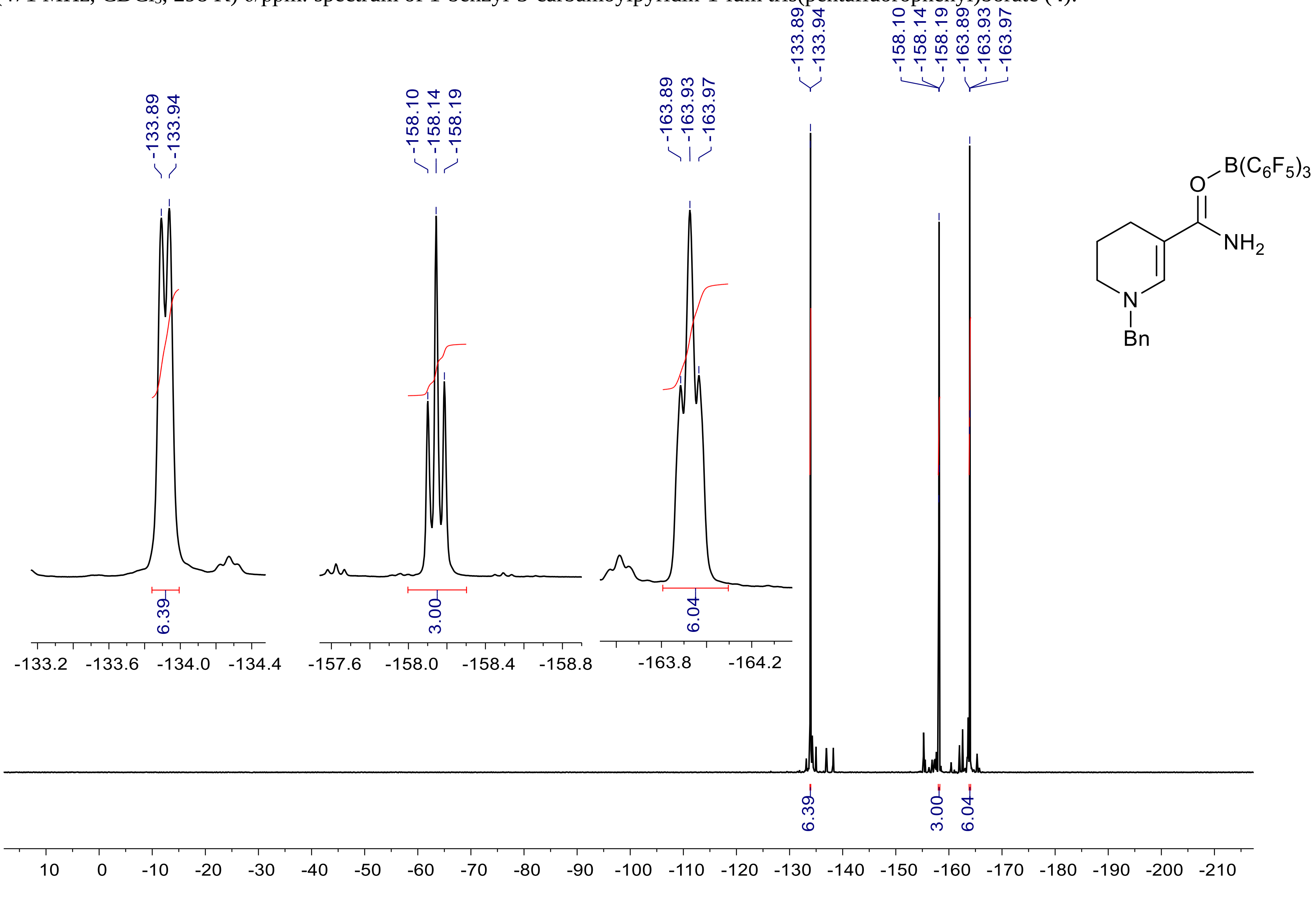

© The Authors under the terms of the Creative Commons Attribution License http://creativecommons.org/licenses/by/3.0/, which permits unrestricted use, provided the original author and source are credited.

^{1}H NMR (500 MHz, DMSO, 298 K) spectrum of 1-benzyl-3-(carbamoyl-d)pyridin-1-ium-*N*-d$_2$-tris(pentafluorophenyl)borate (**5**).

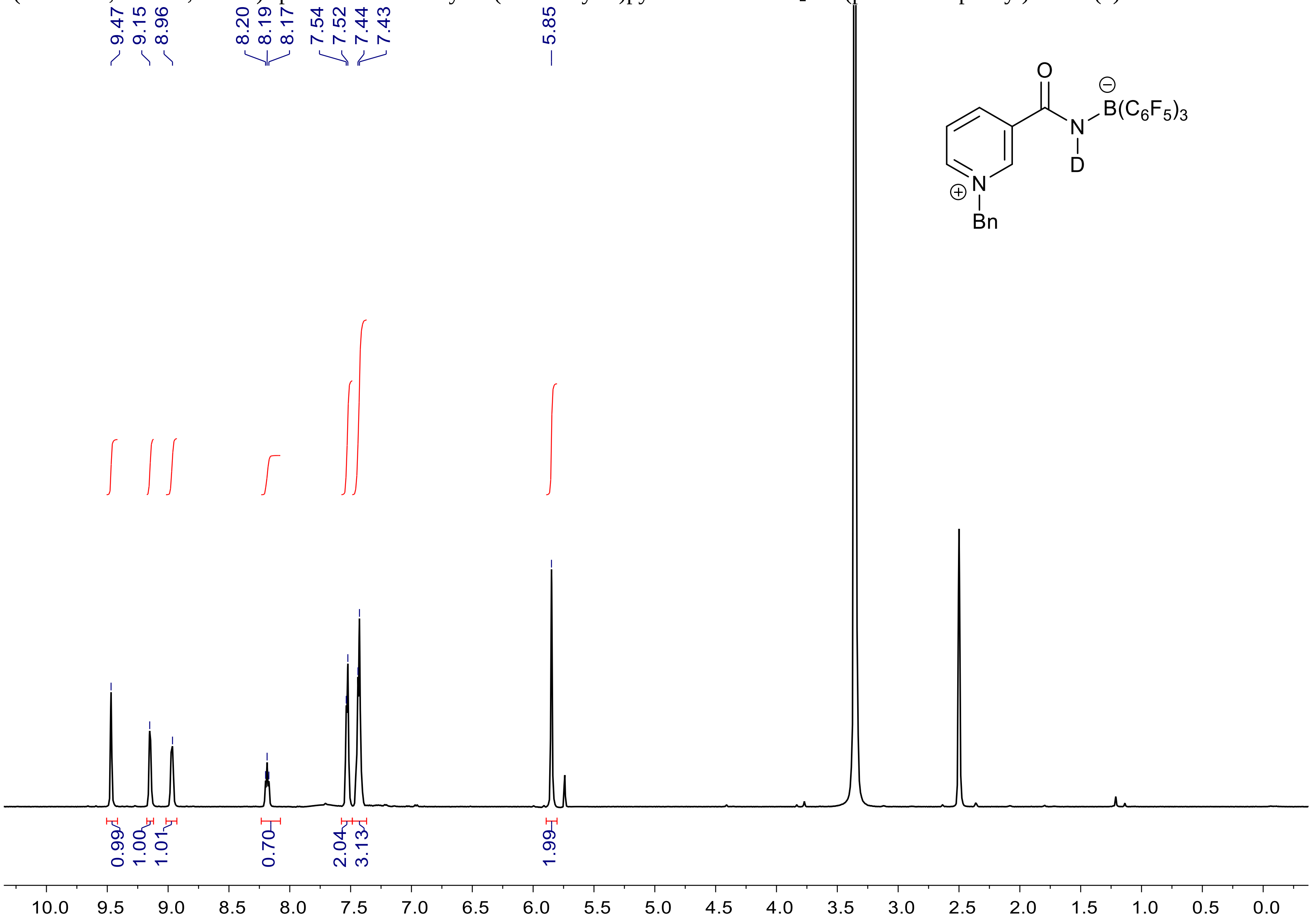

© The Authors under the terms of the Creative Commons Attribution License http://creativecommons.org/licenses/by/3.0/, which permits unrestricted use, provided the original author and source are credited.

^{13}C NMR (126 MHz, $CDCl_3$, 298 K) spectrum of 1-benzyl-3-(carbamoyl-d)pyridin-1-ium-*N*-d_2-tris(pentafluorophenyl)borate (**5**).

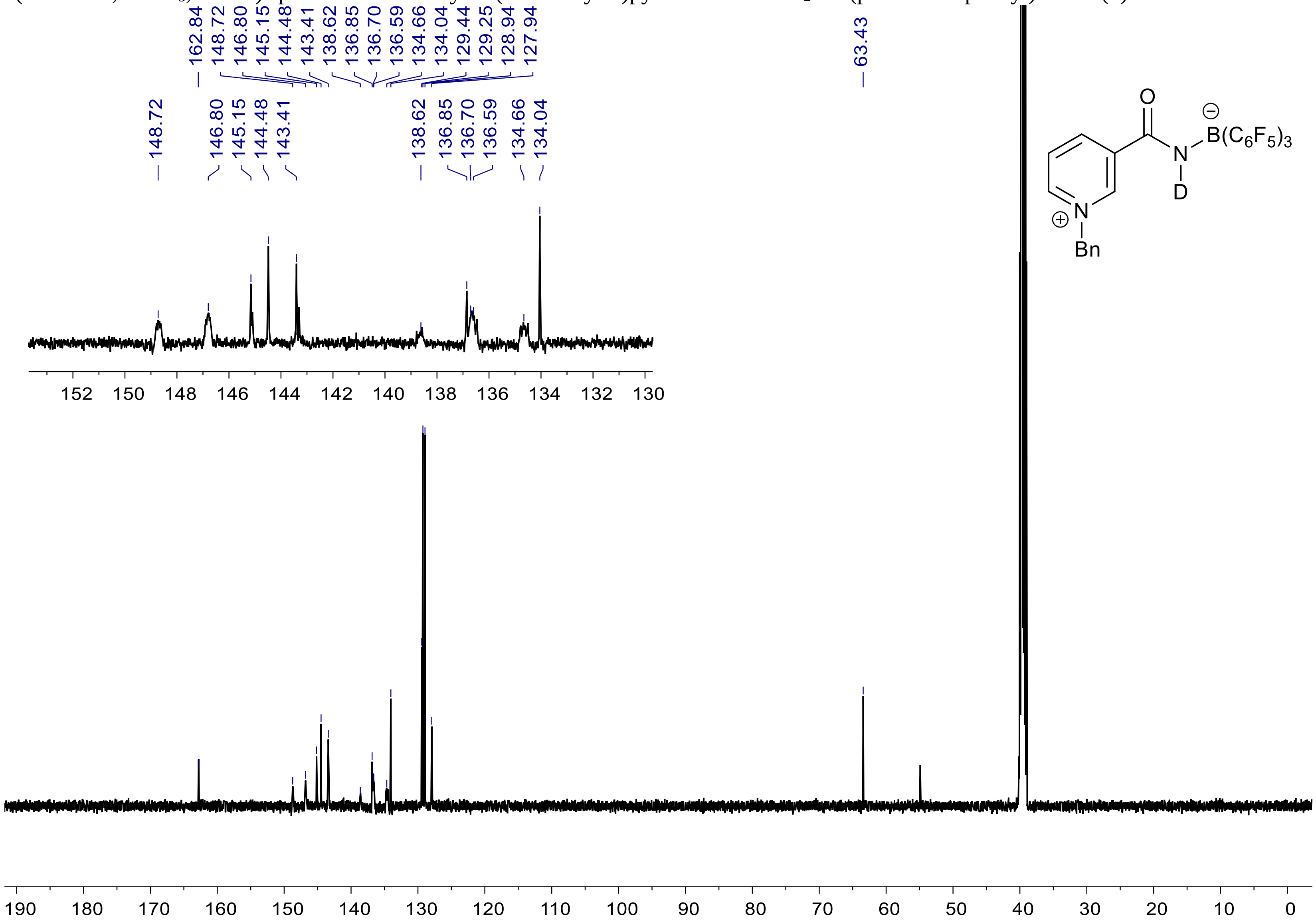

© The Authors under the terms of the Creative Commons Attribution License http://creativecommons.org/licenses/by/3.0/, which permits unrestricted use, provided the original author and source are credited.

^{11}B NMR (160 MHZ, $CDCl_3$, 298 K) δ/ppm: spectrum of 1-benzyl-3-(carbamoyl-d)pyridin-1-ium-*N*-d_2-tris(pentafluorophenyl)borate (**5**).

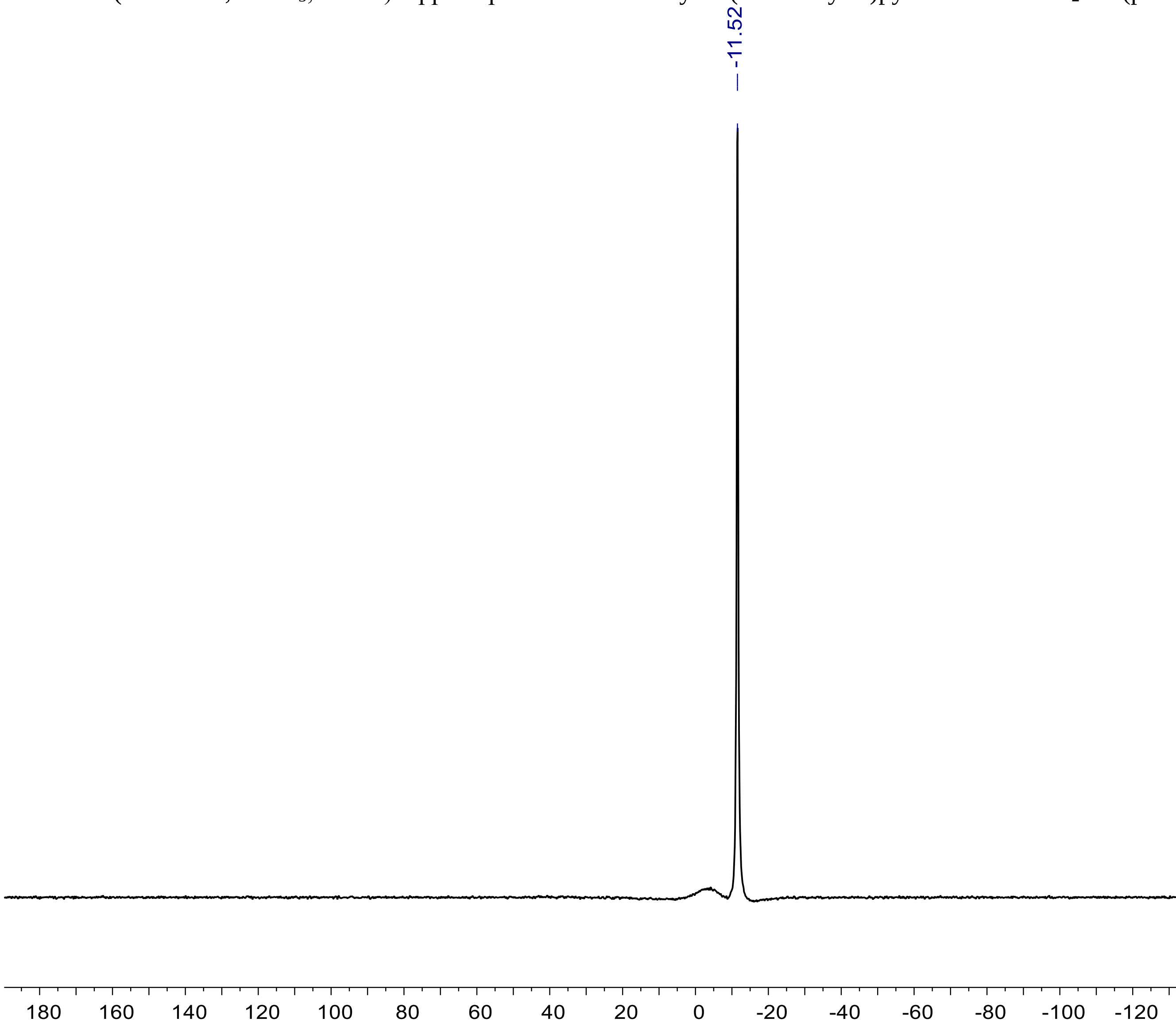

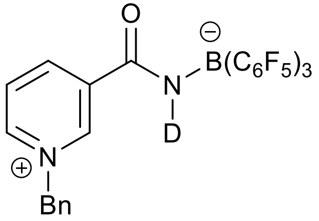

© The Authors under the terms of the Creative Commons Attribution License http://creativecommons.org/licenses/by/3.0/, which permits unrestricted use, provided the original author and source are credited.

^{19}F NMR (471 MHz, $CDCl_3$, 298 K) δ/ppm: spectrum of 1-benzyl-3-(carbamoyl-d)pyridin-1-ium-*N*-d_2-tris(pentafluorophenyl)borate (**5**).

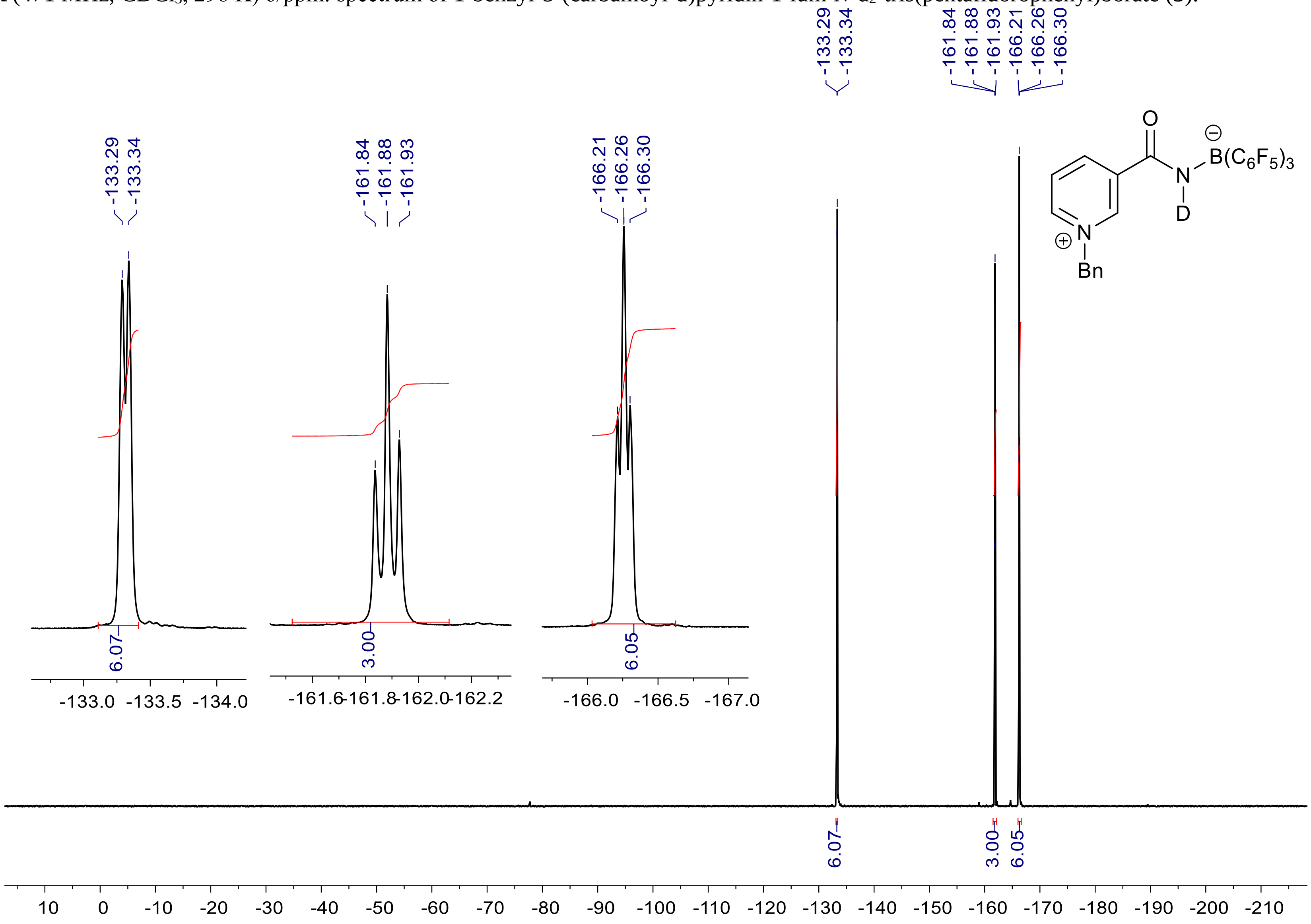

© The Authors under the terms of the Creative Commons Attribution License http://creativecommons.org/licenses/by/3.0/, which permits unrestricted use, provided the original author and source are credited.

^{1}H NMR (500 MHz, $CDCl_3$, 298 K) spectrum of 1-benzyl-1,4,5,6-tetrahydropyridine-5-d-3-carboxamide-*N*,*N*-d_2 tris(pentafluorophenyl) borane adduct (**6**).

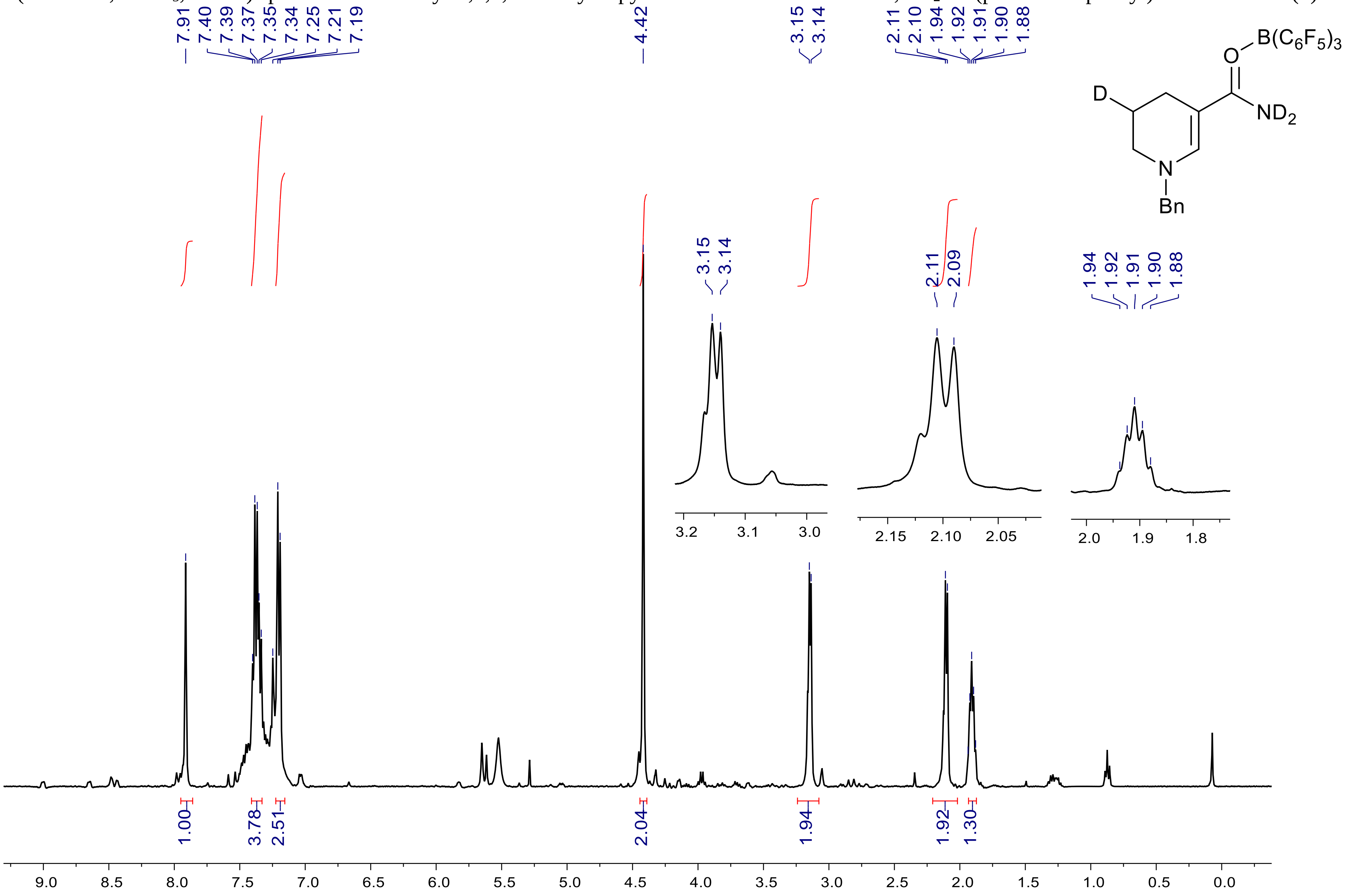

© The Authors under the terms of the Creative Commons Attribution License http://creativecommons.org/licenses/by/3.0/, which permits unrestricted use, provided the original author and source are credited.

Supplementary material for [Author], [Year], [Title], *Phil. Trans. R. Soc. A.* doi: 10.1098/rsta.[paper ID in form xxxx.xxxx e.g. 10.1098/rsta.2014.0049]

^{13}C NMR (126 MHz, $CDCl_3$, 298 K) spectrum of 1-benzyl-1,4,5,6-tetrahydropyridine-5-d-3-carboxamide-*N*,*N*-d_2 tris(pentafluorophenyl) borane adduct (**6**).

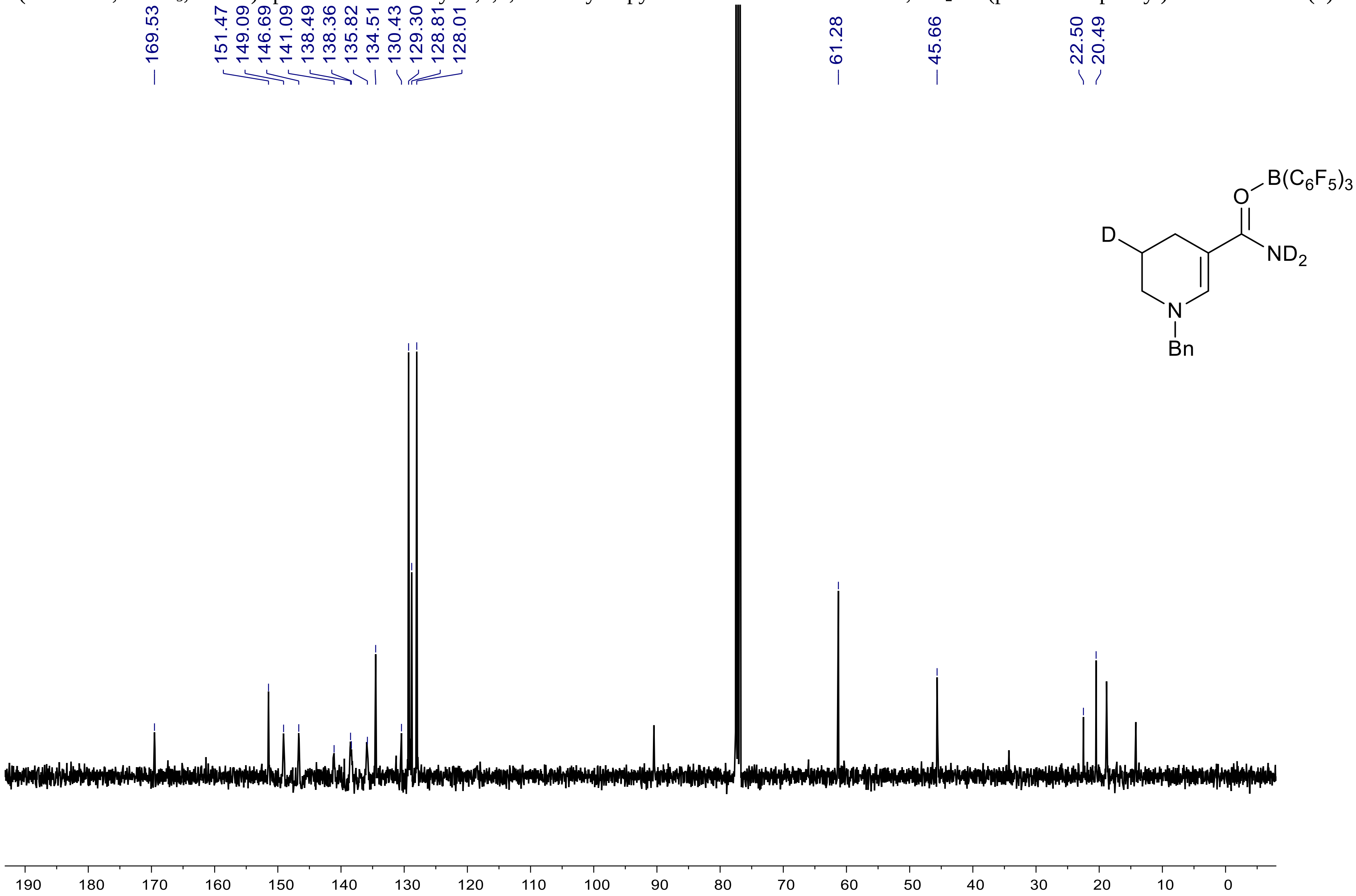

© The Authors under the terms of the Creative Commons Attribution License http://creativecommons.org/licenses/by/3.0/, which permits unrestricted use, provided the original author and source are credited.

^{11}B NMR (160 MHZ, $CDCl_3$, 298 K) spectrum of 1-benzyl-1,4,5,6-tetrahydropyridine-5-d-3-carboxamide-*N*,*N*-d_2 tris(pentafluorophenyl) borane adduct (**6**).

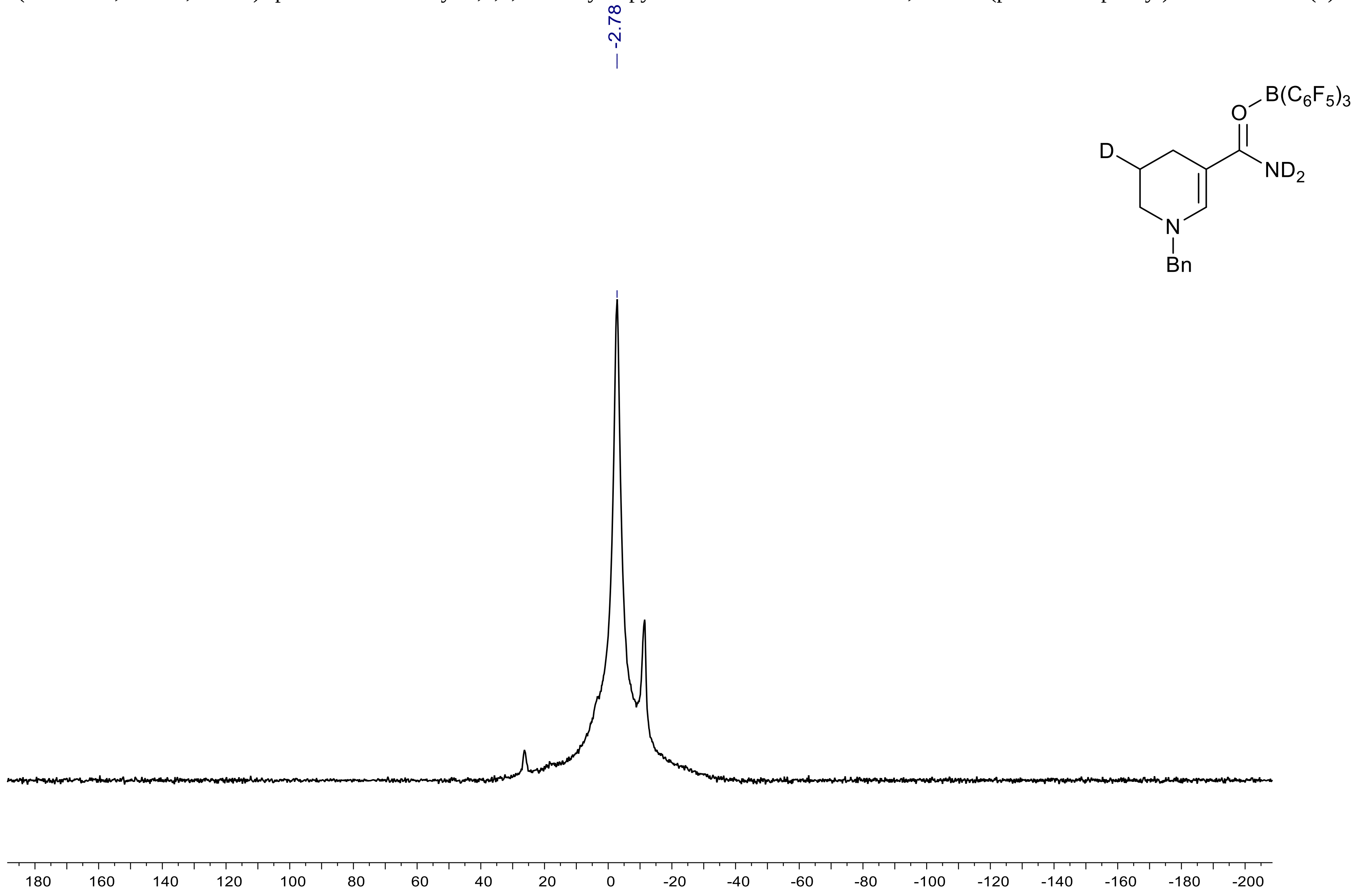

© The Authors under the terms of the Creative Commons Attribution License http://creativecommons.org/licenses/by/3.0/, which permits unrestricted use, provided the original author and source are credited.

^{19}F NMR (471 MHz, $CDCl_3$, 298 K) spectrum of 1-benzyl-1,4,5,6-tetrahydropyridine-5-d-3-carboxamide-*N*,*N*-d$_2$ tris(pentafluorophenyl) borane adduct (**6**).

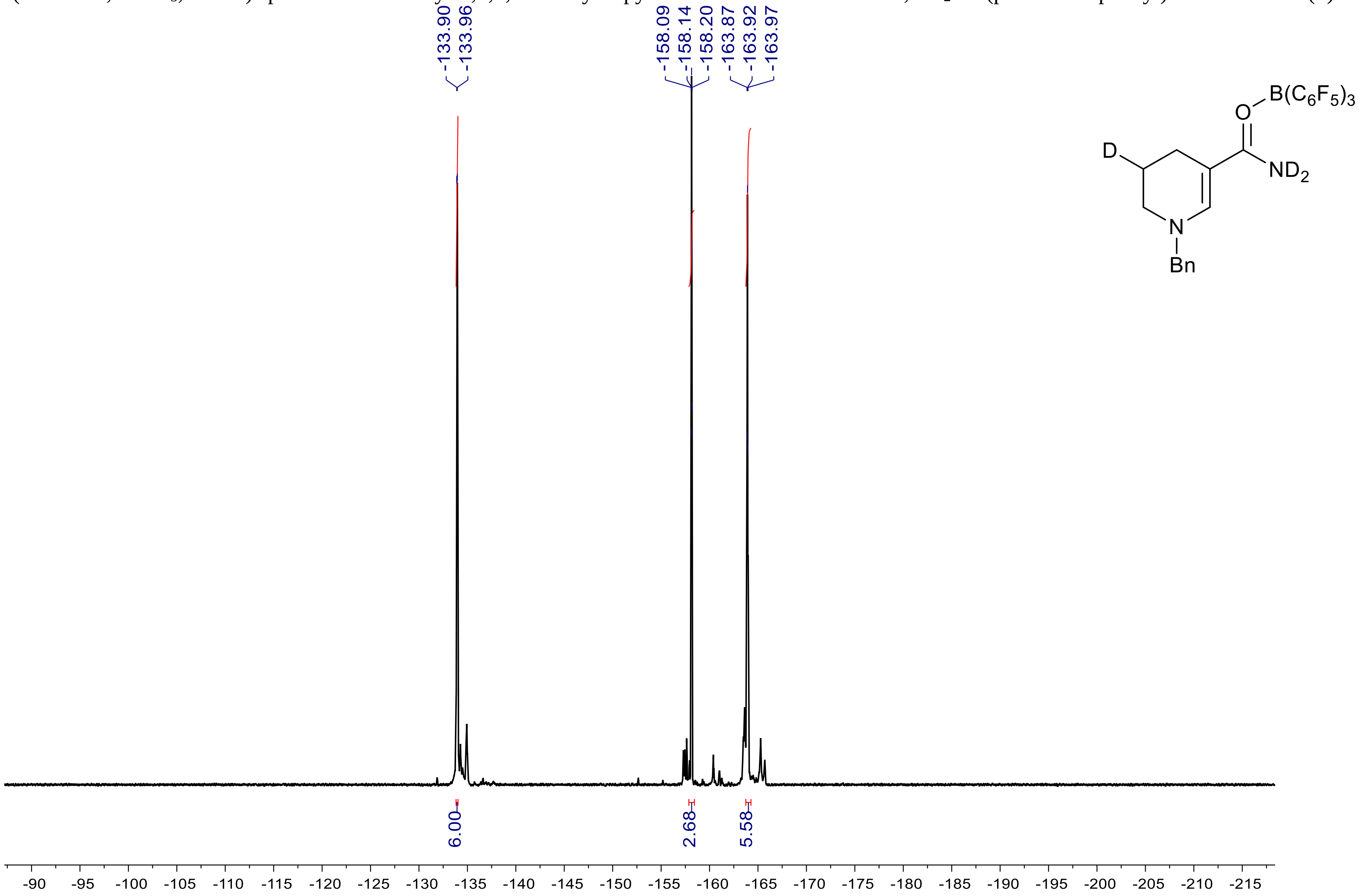

© The Authors under the terms of the Creative Commons Attribution License http://creativecommons.org/licenses/by/3.0/, which permits unrestricted use, provided the original author and source are credited.

^{1}H NMR (500 MHz, DMSO-d_6, 298 K) spectrum of ((1-benzylpyridin-1-ium-3-carbonyl)oxy)tris(pentafluorophenyl)borate (**7**).

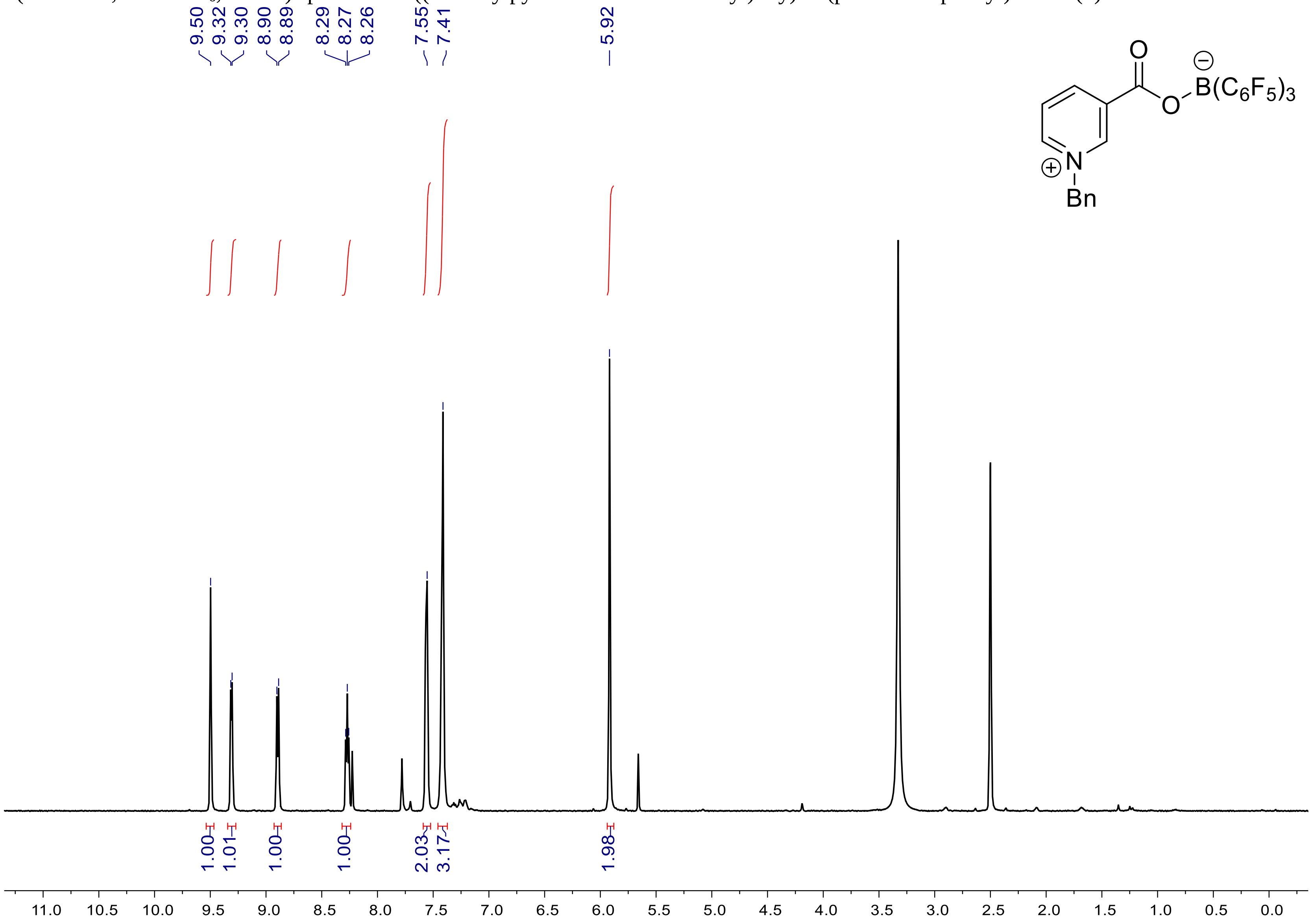

© The Authors under the terms of the Creative Commons Attribution License http://creativecommons.org/licenses/by/3.0/, which permits unrestricted use, provided the original author and source are credited.

^{13}C NMR (126 MHz, DMSO-d$_6$, 298 K) spectrum of ((1-benzylpyridin-1-ium-3-carbonyl)oxy)tris(pentafluorophenyl)borate (**7**).

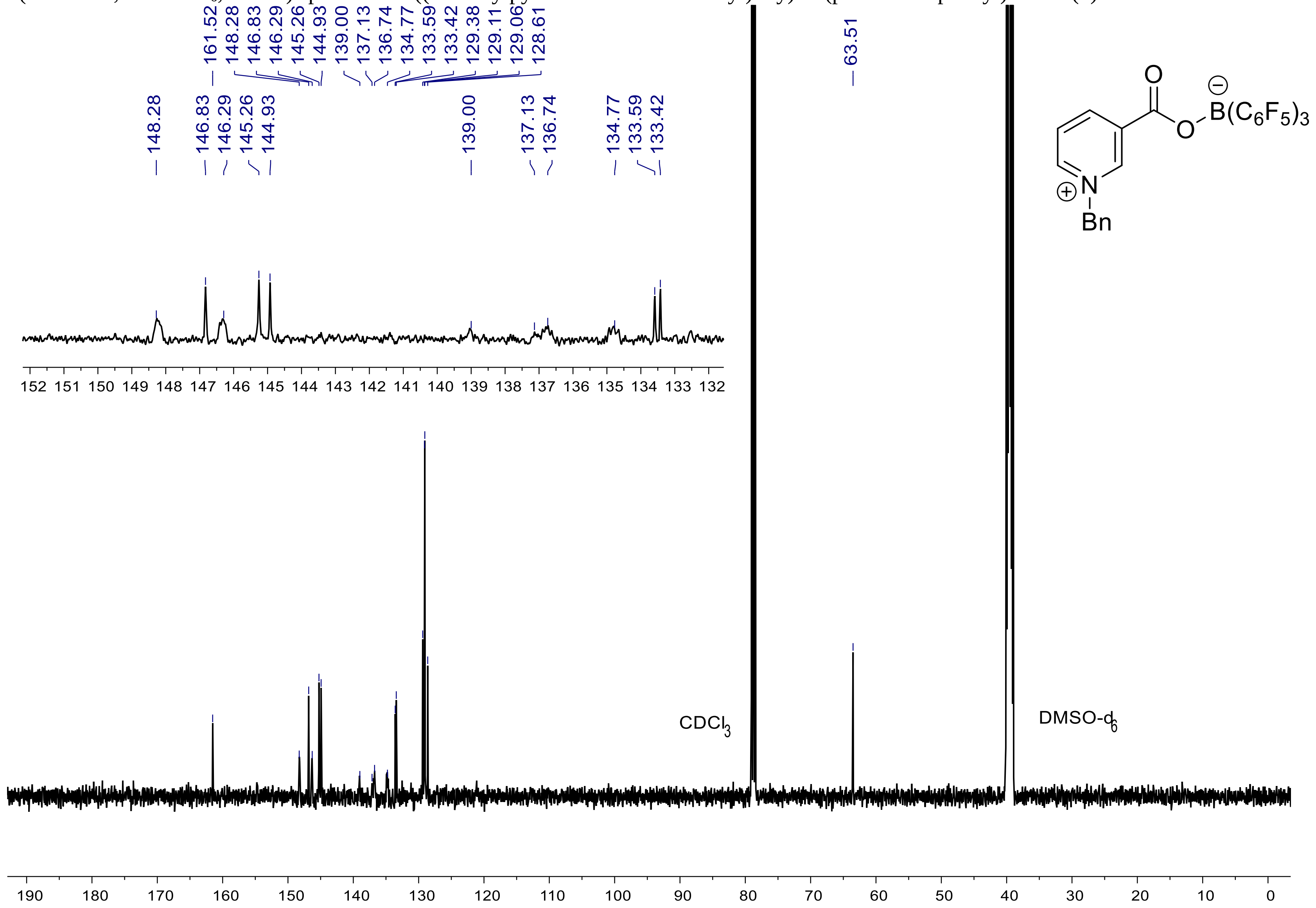

© The Authors under the terms of the Creative Commons Attribution License http://creativecommons.org/licenses/by/3.0/, which permits unrestricted use, provided the original author and source are credited.

^{11}B NMR (160 MHZ, DMSO-d$_6$, 298 K) spectrum of ((1-benzylpyridin-1-ium-3-carbonyl)oxy)tris(pentafluorophenyl)borate (**7**).

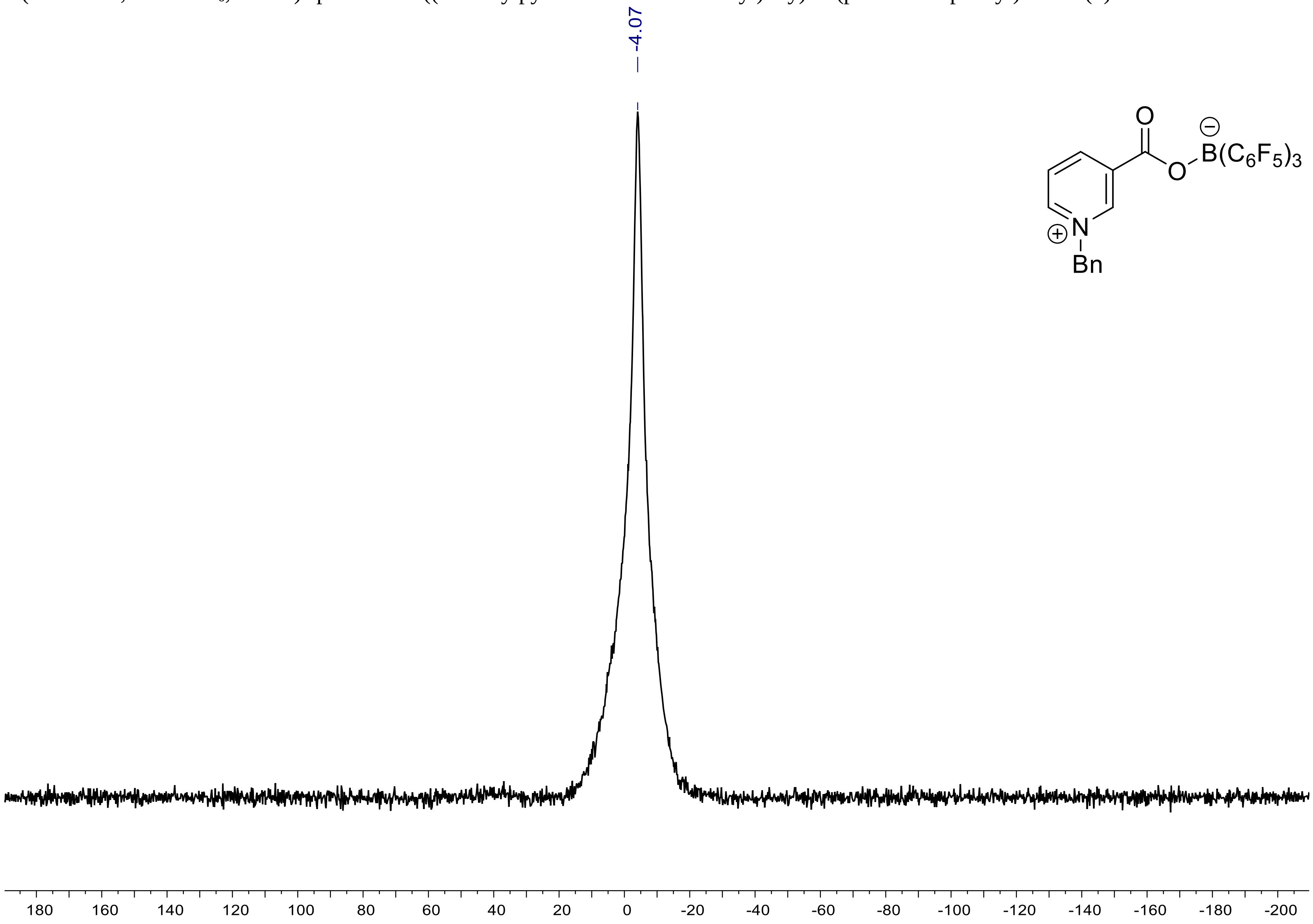

© The Authors under the terms of the Creative Commons Attribution License http://creativecommons.org/licenses/by/3.0/, which permits unrestricted use, provided the original author and source are credited.

^{19}F NMR (471 MHz, DMSO-d_6, 298 K) spectrum of ((1-benzylpyridin-1-ium-3-carbonyl)oxy)tris(pentafluorophenyl)borate (**7**).

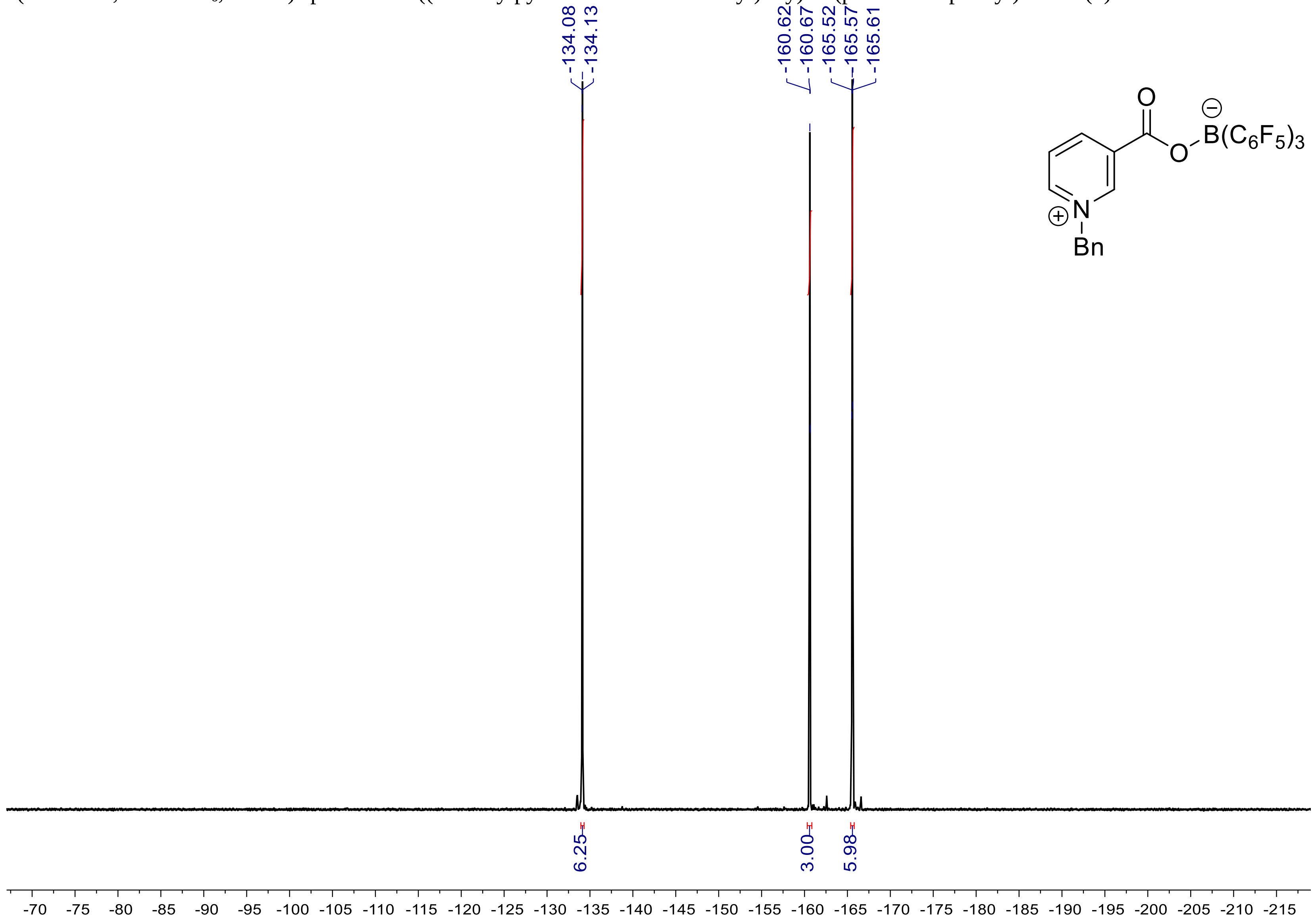

© The Authors under the terms of the Creative Commons Attribution License http://creativecommons.org/licenses/by/3.0/, which permits unrestricted use, provided the original author and source are credited.

5. Theoretical considerations

Geometry optimisations through computational means were undertaken using the B3LYP hybrid functional[5-6] whilst instituting the 6-31G*[7] basis set using Gaussian 09 software[8] based on coordinates of **3** gained from experimental data. Images of these structures were generated through ORTEP-3[9] and Mercury.[10]

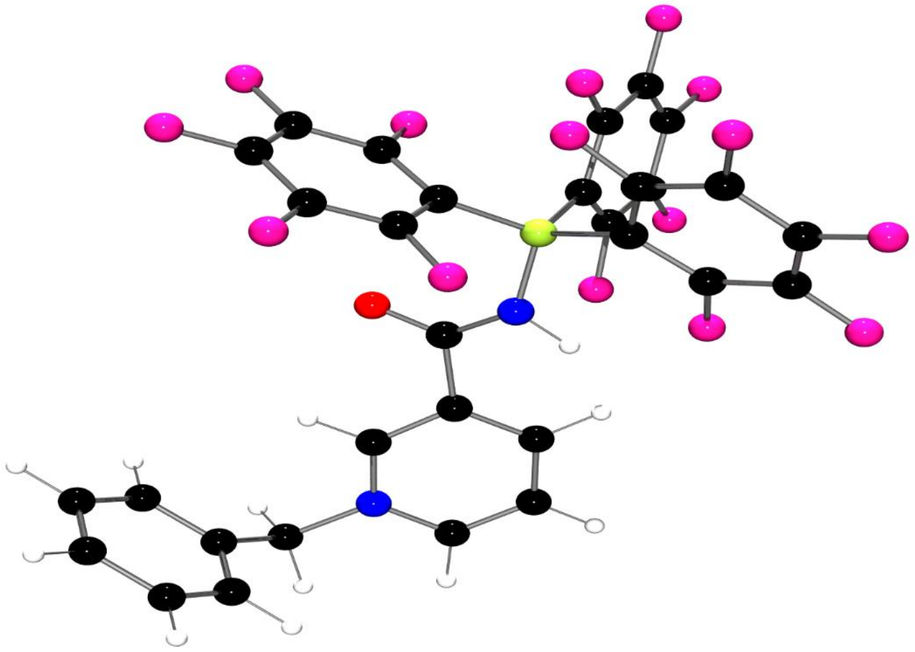

Figure S1. Ball and stick representation of calculated structure.

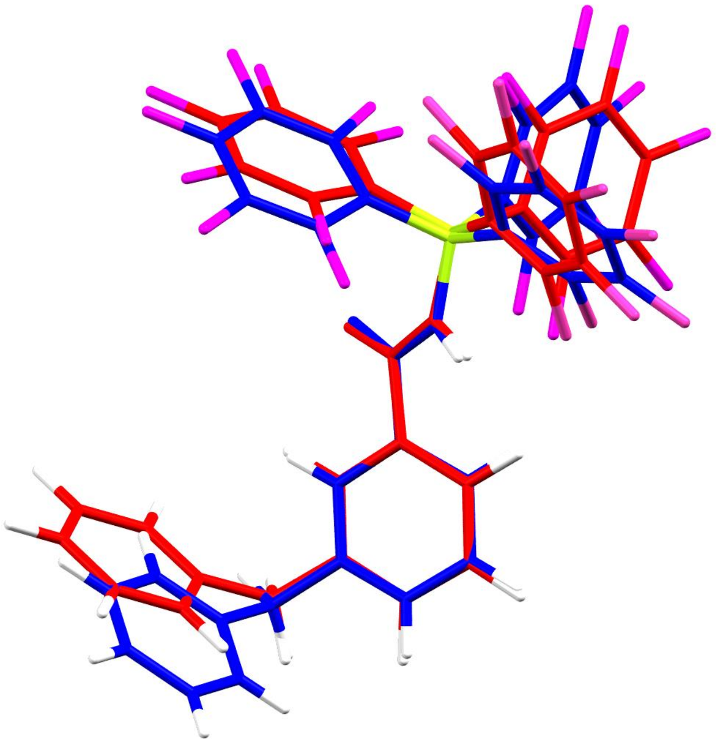

Figure S2. Overlay structure of experimental data (blue) and theoretical structure (red).

© The Authors under the terms of the Creative Commons Attribution License http://creativecommons.org/licenses/by/3.0/, which permits unrestricted use, provided the original author and source are credited.

6. Crystallographic studies.

Single crystals of **3** were grown under an inert atmosphere. Crystallographic studies were undertaken of a single crystal mounted in paratone and studied on an Agilent SuperNova Dual three-circle diffractometer using Cu-Kα radiation and a CCD detector. Measurements were carried out at 150(2) K with temperatures maintained using an Oxford cryostream unless otherwise stated. Data were collected and integrated and data corrected for absorption using a numerical absorption correction based on gaussian integration over a multifaceted crystal model within CrysAlisPro.[3] The structure was solved by direct methods and refined against F^2 within SHELXL-2013.[4] A summary of crystallographic data are available as ESI and the structure deposited with the Cambridge Structural Database (CCDC deposition numbers 1532113). This data can be obtained free of charge from The Cambridge Crystallographic Data Centre via www.ccdc.cam.ac.uk/data_request/cif.

Compound	**3**
Empirical Formula	$C_{31}H_{12}BF_{15}N_2O$, $CHCl_3$
Crystal System	Triclinic
Space Group	*P-1*
a/Å	12.5821(9)
b/Å	12.6326(8)
c/Å	12.9076(8)
α/°	88.937(5)
β/°	79.854(5)
γ/°	66.759(6)
V/Å^3	1852.7(2)
Z	2
T /K	150(2)
D_c/g.cm^{-3}	1.512
Crystal size/mm	0.289 x 0.187 x 0.122
Total data	12937
Unique data	7276
R_{int}	0.0463
$R_1[F^2>2\sigma(F^2)]$	0.0478
wR2 (all data)	0.1409
GoF	1.026
ρ_{min}/ρ_{max}/eÅ^{-3}	-0.778/0.449
CCDC code	1532113

© The Authors under the terms of the Creative Commons Attribution License http://creativecommons.org/licenses/by/3.0/, which permits unrestricted use, provided the original author and source are credited.

Thermal ellipsoid plot of **3**. Ellipsoids shown at 50% probability. Chloroform solvent molecule removed for clarity. C: black, H: white, N: blue, B: yellow-green, F: pink.

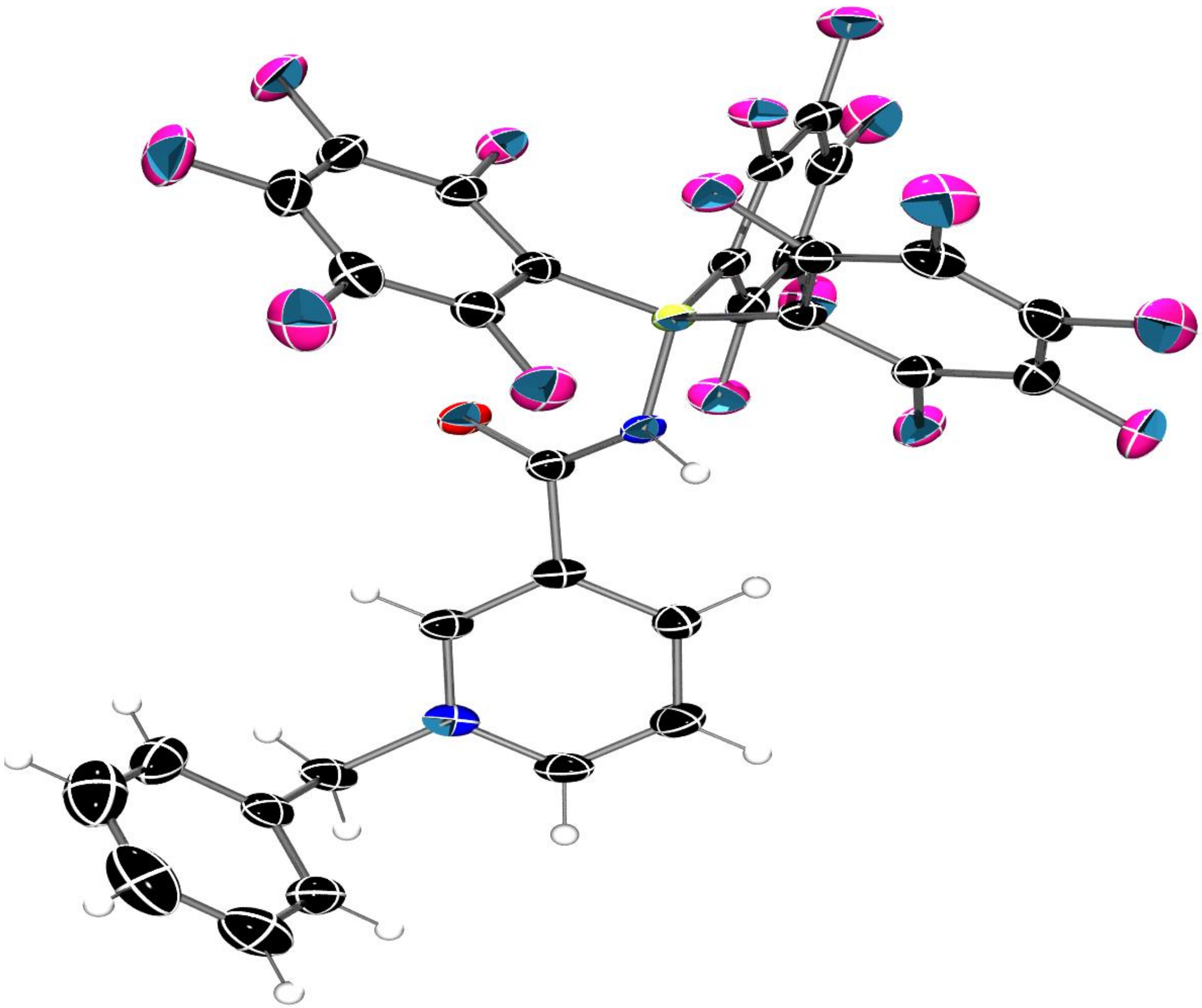

© The Authors under the terms of the Creative Commons Attribution License http://creativecommons.org/licenses/by/3.0/, which permits unrestricted use, provided the original author and source are credited.

7. References:

1. Mohanty AD, Bae C. 2014 Mechanistic analysis of ammonium cation stability for alkaline exchange membrane fuel cells. *J. Mater. Chem. A* **2**, 17314-17320. (doi:10.1039/C4TA03300K).
2. Paul CE, Gargiulo S, Opperman DJ, Lavandera I, Gotor-Fernández V, Gotor V, Taglieber A, Arends IWCE, Hollmann F. 2013 Mimicking Nature: Synthetic Nicotinamide Cofactors for C═C Bioreduction Using Enoate Reductases. *Org. Lett.* ***15***, 180-183. (doi:10.1021/ol303240a).
3. CrysAlisPro, Agilent Technologies, Version 1.171.37.33 (release 27-03-2014 CrysAlis171 .NET).
4. SHELXL-2013, G. M. Sheldrick, University of Göttingen, Germany (2013).
5. Becke AD. 1988 *Phys. Rev. A: At. Mol. Opt. Phys.* ***38***, 3098. (doi: 10.1103/PhysRevA.38.3098).
6. Lee C, Yang W, Parr RG. 1988 *Phys. Rev. A: At. Mol. Opt. Phys.* ***37***, 785. (doi: 10.1103/PhysRevB.37.785).
7. Ditchfield R, Hehre WJ, Pople JA. 1971 *J. Chem. Phys.* ***54***, 724. (doi: 10.1063/1.1674902).
8. Frisch MJ, Trucks GW, Schlegel HB, Scuseria GE, Robb MA, Cheeseman JR, Scalmani G, Barone V, Mennucci B, Petersson GA, Nakatsuji H, Caricato M, Li X, Hratchian HP, Izmaylov AF, Bloino J, Zheng G, Sonnenberg JL, Hada M, Ehara M, Toyota K, Fukuda R, Hasegawa J, Ishida M, Nakajima T, Honda Y, Kitao O, Nakai H, Vreven T, Montgomery Jr. JA, Peralta JE, Ogliaro F, Bearpark MJ, Heyd J, Brothers EN, Kudin KN, Staroverov VN, Kobayashi R, Normand J, Raghavachari K, Rendell AP, Burant JC, Iyengar SS, Tomasi J, Cossi M, Rega N, Millam NJ, Klene M, Knox JE, Cross JB, Bakken V, Adamo C, Jaramillo J, Gomperts R, Stratmann RE, Yazyev O, Austin AJ, Cammi R, Pomelli C, Ochterski JW, Martin RL, Morokuma K, Zakrzewski VG, Voth GA, Salvador P, Dannenberg JJ, Dapprich S, Daniels AD, Farkas Ö, Foresman JB, Ortiz JV, Cioslowski J, Fox DJ, Gaussian, Inc., Wallingford, CT, USA, **2009**.
9. Farrugia L. 2012 WinGX and ORTEP for Windows: an update. *J. Appl. Crystallogr.* ***45***, 849-854. (doi:10.1107/S0021889812029111).
10. Macrae CF, Edgington PR, McCabe P, Pidcock E, Shields GP, Taylor R, Towler M, van de Streek J. 2006 Mercury: visualization and analysis of crystal structures. *J. Appl. Crystallogr.* ***39***, 453-457. (doi:10.1107/S002188980600731X).

© The Authors under the terms of the Creative Commons Attribution License http://creativecommons.org/licenses/by/3.0/, which permits unrestricted use, provided the original author and source are credited.
